# Seasonal Variations in Maternal Provisioning of *Crepidula* *fornicata* (Gastropoda): Fatty Acid Composition of Females, Embryos and Larvae

**DOI:** 10.1371/journal.pone.0075316

**Published:** 2013-09-24

**Authors:** Fanny Leroy, Tarik Meziane, Pascal Riera, Thierry Comtet

**Affiliations:** 1 UPMC Univ Paris 06, UMR 7144, Station Biologique, Roscoff, France; 2 CNRS, UMR 7144, Station Biologique, Roscoff, France; 3 Muséum National d’Histoire Naturelle, UMR BOREA 7208, Committee for Programme and Coordination 53, Paris, France; CSIR- National institute of oceanography, India

## Abstract

Recruitment success of marine invertebrate populations not only depends on the number of recruits but also on their quality which affects their survival. In species characterized by a mixed development (encapsulated embryonic development and release of planktotrophic larvae), the offspring quality depends on both maternal provisioning and larval feeding. Here, we investigated potential changes of maternal provisioning over the whole reproductive period in a gastropod with a mixed development: *Crepidula* *fornicata* . In its introduction area, *C* *. fornicata* reproduces from February to October, which implies that both adults and larvae are exposed to different food availabilities. Maternal provisioning was assessed by measuring the fatty acid (FA) composition of females, encapsulated embryos and larvae, in February, May, July and September 2009. FA are essential resources for the development of embryos and larvae, and are key biomarkers of offspring quality. Our results showed differences in FA composition between muscles, visceral masses, and encapsulated embryos. In particular, FA composition of embryos was similar to that of the visceral mass. Seasonal variations in FA composition were observed: in the middle of the reproductive season (May and July), female tissues and embryos showed a higher proportion of polyunsaturated fatty acids and especially ω3, as compared to the beginning and end of the reproductive season (February and September). This showed that through maternal provisioning the quality of *C* *. fornicata* offspring was higher in the middle of the reproductive season. Whether this would result in an increase of recruitment success and juvenile performance would require further investigations.

## Introduction

In most marine invertebrates, larval life history traits (developmental mode, growth, survival...) are intimately linked to the egg size and nutrient content provided by the mother (e.g. [1-4]). This is not only true for species with lecithotrophic development which mostly rely on maternally-provided resources and do not need to feed, but also for species producing planktotrophic larvae which need to feed in order to complete their development. Whereas lecithotrophic eggs have a greater weight-specific energy content to achieve complete development [5], in planktotrophs, offspring performance depends on both endogenous reserves derived from the parents and exogenous resources provided through larval nutrition [4,6-8]. Assessing the relative contribution of these two sources is of primary importance for the understanding of larval development and juvenile performance. In some species, parental provisioning has been shown to influence larval growth and survival (e.g. [1,6]), being in some cases even more important than larval feeding [8], whereas in some others increase in maternal investment in eggs has a positive effect on larval size but not on larval development at natural food concentrations [9]. Maternal supply to embryos is influenced by numerous factors: mother body size, maternal feeding, and maternal habitat which includes food availability for parents [1,2,6,9-12]. This latter factor is crucial for species with a long reproductive period, with both parents and offspring facing seasonal variations of the environmental conditions, including food abundance and composition. In these species, the offspring condition may thus vary seasonally (e.g. [13]), with potential consequences on dispersal abilities and juvenile performance.

Maternal investment is not only important in terms of quantity, but the biochemical composition of nutrients provided by females is also fundamental for the condition of offspring [14]. In particular, lipids are the major metabolic energy reserve in most marine animals, including marine invertebrate larvae [15]. They play a central role in the embryo development as an energy resource [15-17] and influence the hatching rate, the larval survival and the settlement success of many invertebrate species [18,19]. Fatty acids (FA) are the fundamental structural components of almost all lipid forms [20]. They are known to play a major role in embryogenesis and larval development, especially the polyunsaturated fatty acids (PUFA). In particular, PUFA with a terminal end omega-3 (ω3) are mostly a source of energy (with the exception of the docosahexaenoic acid (DHA; 22:6ω3), which has a role in membrane integrity), and omega-6 (ω6) PUFA are structural components of cell membranes. The PUFA EPA (20:5ω3; eicosapentaenoic acid) and DHA were shown to increase hatching success and larval survival in bivalves [21-24] and gastropods [25]. Therefore, FA composition of eggs and larval tissues may be used to assess the offspring condition. Besides, FA composition of eggs and larvae depends on both parental and larval nutrition [26] and may be considered as trophic biomarkers allowing the identification of assimilated food sources (e.g. [20,27-30]).

**Figure 1:**
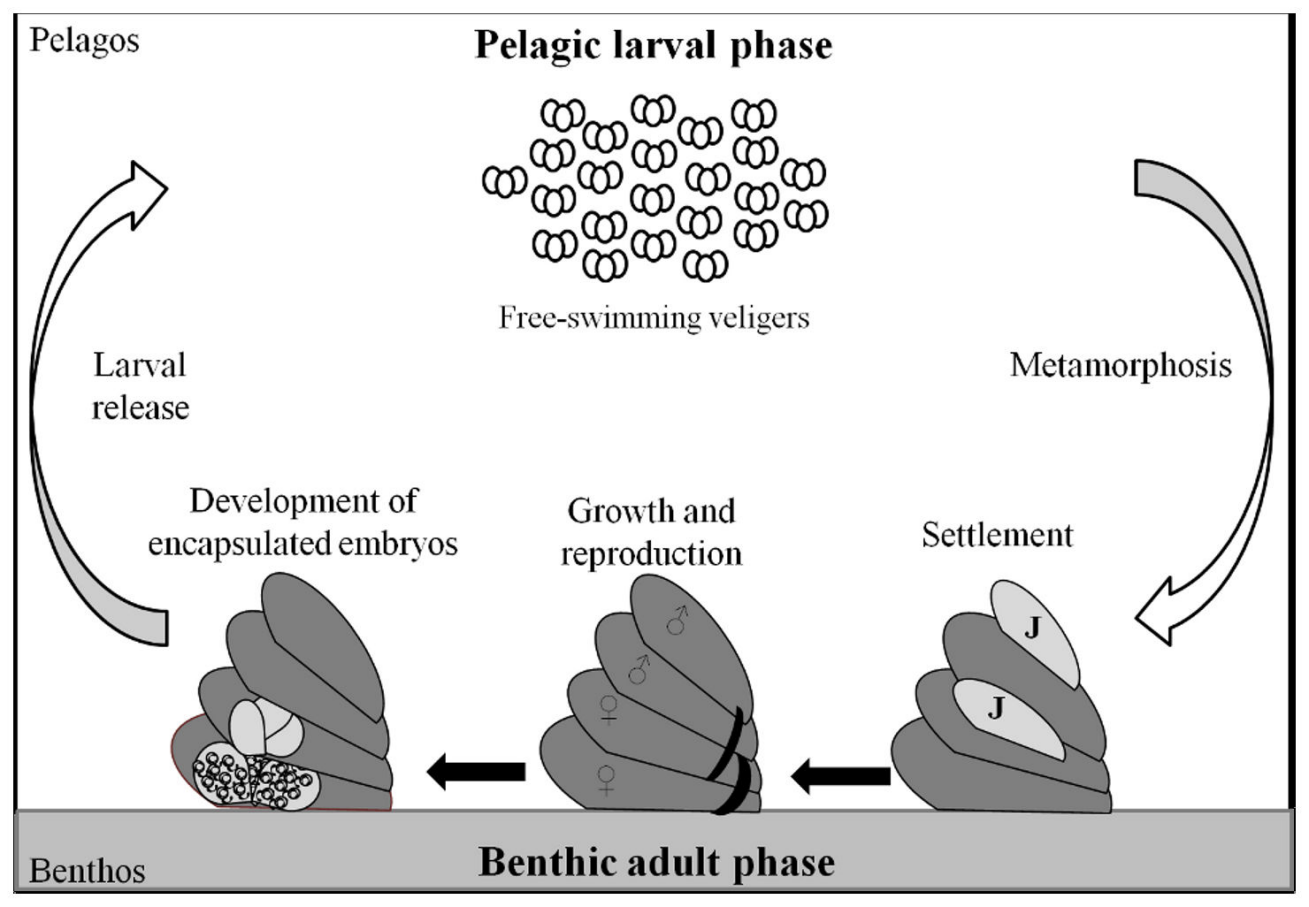
Schematic of the bentho-pelagic life life cycle of *Crepidula* *fornicata* .

In this paper, we investigated the seasonal variations in maternal investment in embryos of the gastropod *Crepidula* *fornicata* Linnaeus 1758 (Calyptraeidae) and its consequences on the quality of released larvae. *C* *. fornicata* broods its embryos for about one month within thin-walled capsules located between the neck and the propodium of the mother, before releasing them as planktotrophic larvae [31,32] (Figure 1). Each female broods between 25 and 75 capsules, each containing 300 to 500 embryos, all at the same developmental stage [33,34]. To our knowledge, in *C* *. fornicata* there is no potential for nutritive exchange between females and their associated broods, and encapsulated embryos have a limited access to external dissolved organic matter [35], so encapsulated embryos mainly or even exclusively rely on resources provided by the mother. Resources mostly consist of yolk [16] and intracapsular albumen [33], investment in the form of extra-embryonic protection (e.g. egg capsules) being low (2-8% of total energy supply in closely-related calyptraeid species [36,37]). Finally, nurse eggs have not been observed in *C* *. fornicata* [38] although cannibalism on damaged eggs or embryos has been reported [39].

Native from the East Coast of North America, *C* *. fornicata* has been introduced in Europe where it has become invasive in many bays and estuaries [40]. In its native and introduced areas, *C* *. fornicata* has a long reproductive period. For example, in the North Coast of France, *C* *. fornicata* reproduces from February to October and larvae are frequently observed from March to November [41-43]. This suggests that resource allocation to offspring may vary along the reproductive period, which is especially important in the context of the introduction of this species, reproduction success being a key feature for successful invasion.

Seasonal variations in maternal investment in embryos of *Crepidula* *fornicata* were investigated by studying the variations of the FA composition of females, encapsulated embryos at four developmental stages, and pelagic larvae during one reproductive season.

## Materials and Methods

### Sample collection

**Figure 2:**
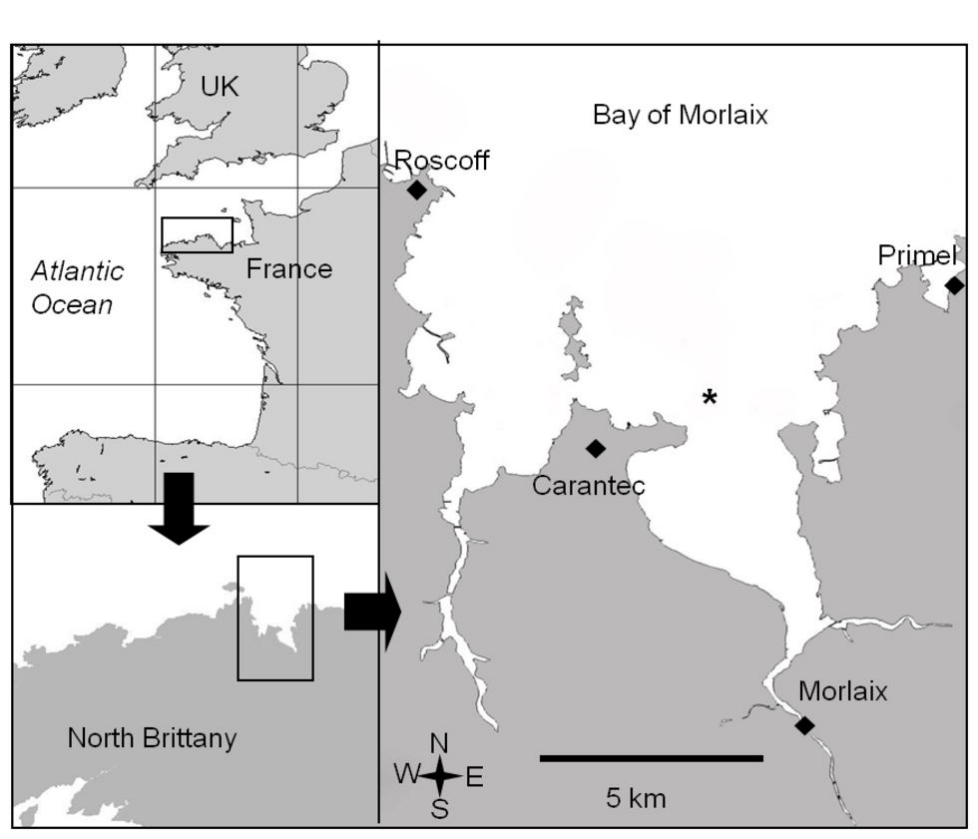
The sampling site. Map showing the location of the sampling site (black cross) in the bay of Morlaix (48°40’ N. 3° 53’W), North Brittany, France.

Stacks of *Crepidula* *fornicata* were collected with a grab in the bay of Morlaix (48°40’ N, 3°53’ W; Fig. 2), Brittany, France. In this species, the reproductive period extends from February to October [41-43]. To cover the whole period, sampling was performed at four occasions in 2009: 26^th^ February, 5^th^ May, 2^nd^ July and 18^th^ September. Individuals were starved in 2-liter tanks filled with filtered seawater (0.45 µm) during 24 hours before tissue and brood sampling, in order to empty the gut. The water was changed three times during this period. Stacks were then opened in order to collect the broods located between the neck and the propodium of the females. Without prior knowledge of changes that might occur in FA composition during embryo development, we first aimed to compare the FA composition of early embryos. Second, because evidencing potential seasonal differences in the FA composition of early embryos would not necessarily imply seasonal differences in the FA composition of released larvae, we investigated the potential changes occurring in subsequent developmental stages, at each sampling period. Using a dissecting microscope four developmental stages were identified according to Chipperfield [44]: early embryo (I; one cell to gastrula; orange to yellow), trochophore (II; ciliated embryo; light yellow), early veliger (III; developing velum, well-formed shell, dark color due to the presence of yolk), and ready-to-hatch veliger (IV; prior to hatching; well-formed shell, well-developed and strongly ciliated velum, translucent color due to the lack of reserves). Embryos were excapsulated and filtered on pre-combusted GF/F filters. Females corresponding to each brood were dissected in two parts: foot (muscle), and visceral mass (which contains viscera and gonad). All samples were freeze-dried and stored at -20°C until fatty acids extraction.

The number of replicates for each developmental stage depended on their availability at time of sampling. Five females were collected in February with their associated broods (all at stage I); twelve were collected both in May and July, with three replicates for each developmental stage; eight females with their associated broods were collected in September with three replicates for early veliger stage, two replicates for early embryo and ready-to-hatch veliger, and one replicate for trochophore. To compare the fatty acid composition of broods at the four sampling occasions, only the early embryos (stage I) were used. Fatty acid contents of the three other developmental stages were then evaluated in May, July and September.

Pelagic larvae of *C* *. fornicata* were collected monthly by horizontal plankton tows (WP2 net, 200 µm mesh size [45]) at the same site from May to October 2009 (six samples: 12^th^ May; 4^th^ June; 1^st^ July; 31^th^ July; 28^th^ August; 6^th^ October). About 300 larvae were identified following Werner [46] and Thiriot-Quiévreux and Scheltema [47], sorted alive and filtered on pre-combusted GF/F filters. In addition, surface and bottom seawater was sampled with a Niskin bottle to collect particulate organic matter (POM). For this purpose, 3 to 4 L of seawater were pre-filtered on 20 and 200 µm mesh size and filtered on pre-combusted GF/F filters. All filters were freeze-dried and stored at -20°C until FA extraction.

No specific permissions were required for sampling at the selected location, as it is not privately-owned or protected. Field sampling did not involve endangered or protected species.

### Fatty acids extraction and analysis

The lipids from female tissues, excapsulated embryos and larvae of *Crepidula* *fornicata* were extracted following the method of Bligh and Dyer [48] modified by Meziane et al. [30]. Briefly, lipids were extracted by homogenization for two minutes followed by ultrasonication for 20 minutes with a mixture of distilled water: methanol: chloroform (1:2:1, v:v:v). The addition of distilled water: chloroform mixture (1:1, v:v) formed an aqueous-organic two-layer system enhanced by centrifugation (3000 rpm, 5 min). The lower chloroform phase, comprising the lipids, was retained and concentrated by rotary evaporation. The lipid extracts were fully dried under nitrogen and then saponified under reflux (1h30, 80°C) with a 2 M NaOH solution in methanol and distilled water (2:1,v:v). Saponification and methylation were carried out according to Meziane and Tsuchiya [49] in order to collect total FA. After this extraction step, FAME (Fatty Acid Methyl Esters) were separated and quantified by gas chromatography (Varian, 3800) equipped with a flame ionization detector. In order to allow the calculation of fatty acids concentration after analysis by gas chromatography, a defined amount of an internal standard (23: 0 FA) was added into the samples before extraction. Hydrogen was used as a carrier gas. At the injection time, the oven temperature was 60°C. It was raised to 150°C at 40°C min^-1^, and remained at this temperature for three minutes. Then the temperature increased at 240°C at the rate of 3°C min^-1^ and remained constant again for three minutes. Most FAME peaks were identified by comparing their retention times with those of standards (Supelco). When needed, some peaks of FA were identified with gas chromatography coupled with mass spectrometry (ThermoFinnigan TRACE DSQ GC-MS).

Fatty acids were designed as *X*:*Y*ω*Z*, where *X* is the number of carbon atoms, *Y* is the number of double bonds and *Z* is the position of the ultimate double bond from the terminal methyl group.

The concentration of each FA (*C*_*FA*_) was calculated according to Schomburg [50]:

$$C_{FA}=(A_{S}/A_{IS})\times (C_{IS}/W_{S})$$

Where *A*_*S*_ was the peak area of a given fatty acid on the chromatogram, *A*_*IS*_ was the peak area of the internal standard, *C*_*IS*_ was the quantity of the internal standard (23: 0) (mg), *W*_*S*_ was the dry weight of the sample (g), and *C*_*FA*_ was expressed in mg_FA_ / g_dry weight_.

To study the changes in FA composition during embryonic development (from stage I to stage IV), we only considered the FA relative abundances (%). Although FA contents were measured for early embryos, they were not estimated in shelled embryos (stage III and IV) because dry weight included shell weight, thus leading to underestimated FA concentrations. Thus, a potential decrease in FA concentration might be only due to an increase in shell weight.

### Statistical Analysis

Data were expressed as the mean ± standard deviation. The non-parametric Kruskall-Wallis test [51] was used to test for differences in the relative abundances of the FA classes in the different tissues and embryos between the four occasions. When significant differences were observed, the Student-Newman-Keuls (SNK) post-hoc test was applied.

The PRIMER software [52] was used for multivariate analyses. The data matrices (fatty acid contributions (% of total fatty acids) in different groups (foot, visceral mass, embryo (stage I to IV), larva and POM) and at different sampling dates) were used to create triangular similarity matrices, based on the Bray-Curtis similarity coefficient, followed by non-metric multidimensional scaling (n-MDS) and hierarchical clustering analyses. Stress values inferior to 0.2 were considered robust following the recommendation of Clarke [53]. No transformation was performed on the data. Differences in fatty acid compositions between the different sampling occasions and the different groups were tested using a one-way analysis of similarity (ANOSIM). The statistical test (R statistic and associated *p*-value), was computed with 5000 permutations [52]. One-way ANOSIM was applied to groups with more than four replicates. When differences (between sampling dates, developmental stages, or groups) in the fatty acid composition were detected, similarity of percentages (SIMPER, a module of PRIMER) analyses were used to determine which fatty acids drive the observed differences.

## Results

### Seasonal variations of FA composition in female tissues and encapsulated embryos (stage I)

45 individual FA were identified in female tissues, and 43 in stage I embryos (Tables 1 and 2). The major FA were polyunsaturated FA (PUFA; 41.8 to 54.0%), followed by saturated FA (SFA; 20.2 to 32.0%), monounsaturated FA (MUFA; 16.0 to 20.8%), branched FA (BFA; 4.0 to 8.3%) and other FA (1.4 to 3.8%). The PUFA 20:5ω3, 22:6ω3, 20:4ω6, 22:5ω3 and 18:4ω3, the SFA 16: 0, 18: 0 and 14: 0, and the MUFA 16:1ω7, 18:1ω7, 18:1ω9, and 20:1ω9 were the most abundant FA. At each sampling date, FA composition of foot, visceral mass and embryos were statistically different (ANOSIM, p<0.01). N-MDS analysis (data not shown) further showed that embryos had a FA composition closer to that of the visceral mass.

**Table 1:** Relative contribution (%) of fatty acids in female tissues (foot and visceral mass) and embryos at stage I of *Crepidula* *fornicata* .

**FA**	**Foot**								**Visceral mass**								**Embryos at stage I**							
	**February**		**May**		**July**		**September**		**February**		**May**		**July**		**September**		**February**		**May**		**July**		**September**	
Saturated																								
12:0	0.00 ± 0.0		0.01 ± 0.0		0.03 ± 0.1		0.05 ± 0.1		0.00 ± 0.0		0.00 ± 0.0		0.00 ± 0.0		0.00 ± 0.0		0.00 ± 0.0		0.00 ± 0.0		0.00 ± 0.0		0.00 ± 0.0	
14:0	1.17 ± 0.2		2.04 ± 0.4		2.84 ± 0.3		2.72 ± 0.4		1.27 ± 0.1		2.20 ± 0.3		2.95 ± 0.5		2.28 ± 0.6		2.17 ± 0.3		2.48 ± 0.2		3.38 ± 0.1		3.08 ± 0.2	
15:0	0.42 ± 0.1		0.55 ± 0.3		0.72 ± 0.1		0.65 ± 0.1		0.60 ± 0.0		0.51 ± 0.1		0.57 ± 0.1		0.57 ± 0.1		0.64 ± 0.1		0.47 ± 0.0		0.58 ± 0.0		0.63 ± 0.0	
16:0	7.36 ± 0.6		10.46 ± 2.3		12.65 ± 1.0		13.30 ± 1.2		10.86 ± 0.6		16.15 ± 1.7		17.70 ± 2.1		14.03 ± 2.1		17.39 ± 1.2		19.86 ± 0.3		20.85 ± 0.6		20.61 ± 0.4	
17:0	1.23 ± 0.3		1.07 ± 0.2		1.29 ± 0.1		1.26 ± 0.1		1.47 ± 0.1		1.00 ± 0.1		1.05 ± 0.1		1.12 ± 0.1		1.51 ± 0.2		0.91 ± 0.0		1.04 ± 0.1		1.30 ± 0.1	
18:0	8.44 ± 0.4		8.16 ± 0.7		8.33 ± 0.5		8.00 ± 0.4		7.23 ± 0.2		6.64 ± 0.5		6.28 ± 0.4		6.18 ± 0.4		5.93 ± 1.0		5.95 ± 0.7		5.38 ± 0.3		5.60 ± 0.7	
19:0	0.00 ± 0.0		0.05 ± 0.1		0.11 ± 0.0		0.12 ± 0.0		0.04 ± 0.1		0.03 ± 0.0		0.04 ± 0.1		0.00 ± 0.0		0.12 ± 0.0		0.00 ± 0.0		0.00 ± 0.0		0.00 ± 0.0	
20:0	1.34 ± 1.2		1.14 ± 1.0		0.67 ± 0.4		1.00 ± 0.4		0.44 ± 0.4		0.64 ± 0.5		0.24 ± 0.3		0.38 ± 0.2		0.38 ± 0.4		0.46 ± 0.2		0.16 ± 0.1		0.08 ± 0.1	
22:0	0.21 ± 0.1		0.17 ± 0.1		0.20 ± 0.1		0.23 ± 0.0		0.07 ± 0.1		0.13 ± 0.2		0.11 ± 0.1		0.02 ± 0.1		0.00 ± 0.0		0.40 ± 0.1		0.07 ± 0.1		0.09 ± 0.1	
24:0	0.00 ± 0.0		0.00 ± 0.0		0.00 ± 0.0		0.00 ± 0.0		0.00 ± 0.0		0.09 ± 0.3		0.00 ± 0.0		0.00 ± 0.0		0.00 ± 0.0		0.00 ± 0.0		0.00 ± 0.0		0.00 ± 0.0	
Sum %	20.17 ± 0.7		23.66 ± 2.6		26.84 ± 1.3		27.31 ± 1.3		21.97 ± 1.6		27.38 ± 3.8		28.93 ± 3.6		24.57 ± 3.5		28.14 ± 0.4		30.53 ± 0.8		31.46 ± 0.6		31.40 ± 0.9	
Monounsaturated																								
15:1	0.58 ± 0.1		0.44 ± 0.2		0.40 ± 0.2		0.21 ± 0.1		0.27 ± 0.0		0.11 ± 0.1		0.14 ± 0.1		0.18 ± 0.2		0.04 ± 0.1		0.07 ± 0.1		0.04 ± 0.1		0.07 ± 0.1	
16:1ω5	0.11 ± 0.2		0.98 ± 0.6		1.49 ± 0.7		2.21 ± 0.3		0.02 ± 0.0		0.53 ± 0.5		0.59 ± 0.6		0.45 ± 0.3		0.03 ± 0.1		0.00 ± 0.0		0.09 ± 0.1		0.10 ± 0.1	
16:1ω7	1.86 ± 0.7		2.35 ± 0.8		2.52 ± 0.2		2.45 ± 0.2		2.08 ± 0.3		3.83 ± 0.5		4.01 ± 0.5		2.30 ± 1.0		3.38 ± 0.1		4.29 ± 0.1		4.35 ± 0.1		3.84 ± 0.1	
16:1ω9	0.18 ± 0.2		0.49 ± 0.6		0.42 ± 0.1		0.38 ± 0.0		0.51 ± 0.0		0.39 ± 0.1		0.44 ± 0.1		0.61 ± 0.6		0.62 ± 0.1		0.51 ± 0.0		0.66 ± 0.0		0.60 ± 0.0	
17:1	4.17 ± 1.5		2.26 ± 1.3		2.09 ± 1.1		0.85 ± 0.6		3.56 ± 1.0		1.55 ± 1.1		1.76 ± 1.2		3.10 ± 1.4		0.91 ± 0.4		0.62 ± 0.5		0.62 ± 0.4		1.05 ± 0.3	
18:1ω11	0.16 ± 0.1		0.18 ± 0.1		0.26 ± 0.0		0.28 ± 0.1		0.16 ± 0.1		0.29 ± 0.1		0.28 ± 0.0		0.20 ± 0.1		0.20 ± 0.1		0.33 ± 0.1		0.36 ± 0.1		0.28 ± 0.0	
18:1ω5	0.11 ± 0.0		0.17 ± 0.1		0.22 ± 0.0		0.23 ± 0.0		0.30 ± 0.0		0.23 ± 0.1		0.21 ± 0.0		0.16 ± 0.1		0.32 ± 0.1		0.27 ± 0.0		0.20 ± 0.0		0.24 ± 0.0	
18:1ω7	1.43 ± 0.2		2.30 ± 0.4		2.28 ± 0.3		2.01 ± 0.2		2.11 ± 0.3		3.56 ± 0.4		3.44 ± 0.4		2.49 ± 0.4		3.72 ± 0.4		4.96 ± 0.3		4.32 ± 0.2		4.31 ± 0.1	
18:1ω9	4.53 ± 0.5		4.75 ± 0.7		4.65 ± 0.7		5.08 ± 0.5		3.55 ± 0.2		2.83 ± 0.5		2.77 ± 0.4		2.98 ± 0.2		3.32 ± 0.2		2.29 ± 0.2		2.36 ± 0.2		2.93 ± 0.0	
20:1ω9	5.21 ± 0.5		4.52 ± 0.4		5.38 ± 0.4		5.96 ± 0.3		4.37 ± 0.4		4.13 ± 0.3		4.05 ± 0.6		5.13 ± 0.7		3.86 ± 0.6		3.13 ± 0.3		2.58 ± 0.1		3.28 ± 0.2	
20:1	1.59 ± 0.4		1.31 ± 0.3		1.08 ± 0.2		1.00 ± 0.2		1.67 ± 0.2		1.02 ± 0.8		0.73 ± 0.1		0.81 ± 0.1		1.25 ± 0.2		0.64 ± 0.0		0.66 ± 0.0		0.65 ± 0.0	
Sum %	19.93 ± 1.2		19.74 ± 1.5		20.79 ± 0.8		20.67 ± 1.0		18.61 ± 2.6		18.46 ± 4.3		18.40 ± 4.0		18.41 ± 4.9		17.65 ± 1.0		17.10 ± 0.4		16.22 ± 0.6		17.35 ± 0.3	
Branched																								
15:0*a*	0.00 ± 0.0		0.31 ± 0.2		0.44 ± 0.2		0.60 ± 0.1		0.00 ± 0.0		0.10 ± 0.1		0.09 ± 0.1		0.04 ± 0.1		0.01 ± 0.0		0.00 ± 0.0		0.00 ± 0.0		0.00 ± 0.0	
15:0*i*	0.05 ± 0.1		0.48 ± 0.3		0.69 ± 0.2		0.93 ± 0.2		0.11 ± 0.1		0.17 ± 0.1		0.24 ± 0.2		0.24 ± 0.1		0.23 ± 0.0		0.06 ± 0.0		0.19 ± 0.0		0.28 ± 0.1	
16:0*i*	0.59 ± 0.1		0.45 ± 0.2		0.65 ± 0.1		0.65 ± 0.1		0.88 ± 0.1		0.54 ± 0.1		0.72 ± 0.1		0.69 ± 0.1		1.25 ± 0.1		0.58 ± 0.1		0.93 ± 0.1		1.06 ± 0.0	
17:0*a*	1.32 ± 0.2		1.46 ± 0.2		1.57 ± 0.2		1.68 ± 0.2		1.72 ± 0.2		1.68 ± 0.2		1.36 ± 0.2		1.39 ± 0.1		2.39 ± 0.2		1.64 ± 0.2		1.59 ± 0.1		1.92 ± 0.1	
17:0*i*	2.51 ± 0.5		2.41 ± 0.4		2.80 ± 0.4		2.77 ± 0.4		3.10 ± 0.4		2.12 ± 0.2		2.44 ± 0.2		2.52 ± 0.3		4.38 ± 0.5		2.26 ± 0.3		2.96 ± 0.2		3.69 ± 0.1	
Sum %	4.48 ± 0.6		5.12 ± 1.0		6.16 ± 0.9		6.62 ± 0.7		5.81 ± 0.7		4.61 ± 0.7		4.85 ± 0.7		4.89 ± 0.7		8.27 ± 0.8		4.53 ± 0.5		5.68 ± 0.3		6.95 ± 0.2	
Polyunsaturated																								
16:2		2.13 ± 0.4		1.82 ± 0.5		2.01 ± 0.4		1.82 ± 0.3		1.04 ± 0.1		0.59 ± 0.2		0.59 ± 0.2		0.73 ± 0.2		0.17 ± 0.1		0.00 ± 0.0		0.00 ± 0.0		0.00 ± 0.0
16:3		2.90 ± 2.9		3.00 ± 1.9		2.40 ± 1.0		3.73 ± 0.8		1.05 ± 1.0		1.78 ± 1.3		1.20 ± 1.0		1.47 ± 0.7		1.12 ± 0.8		0.95 ± 0.5		0.93 ± 0.3		0.66 ± 0.2
18:2ω6		3.13 ± 0.3		3.22 ± 0.4		3.47 ± 0.3		3.57 ± 0.8		2.78 ± 0.3		2.39 ± 0.4		2.67 ± 0.4		2.82 ± 0.4		2.13 ± 0.2		1.50 ± 0.1		1.86 ± 0.1		1.98 ± 0.0
18:3ω3		0.88 ± 0.1		0.96 ± 0.1		0.93 ± 0.1		0.94 ± 0.1		1.06 ± 0.1		1.18 ± 0.1		1.26 ± 0.2		1.10 ± 0.1		1.56 ± 0.2		1.49 ± 0.0		1.71 ± 0.1		1.49 ± 0.1
18:3ω6		0.10 ± 0.1		0.04 ± 0.1		0.10 ± 0.0		0.11 ± 0.0		0.03 ± 0.0		0.09 ± 0.1		0.19 ± 0.1		0.12 ± 0.1		0.19 ± 0.0		0.59 ± 0.6		0.26 ± 0.0		0.32 ± 0.0
18:4ω3		1.69 ± 0.3		2.93 ± 0.7		2.50 ± 0.5		1.98 ± 0.3		2.16 ± 0.4		4.37 ± 0.7		3.71 ± 0.6		3.72 ± 0.4		4.10 ± 0.7		6.05 ± 0.3		5.21 ± 0.4		4.70 ± 0.1
20:2		3.09 ± 0.4		2.39 ± 0.3		2.23 ± 0.3		2.12 ± 0.3		2.76 ± 0.4		1.68 ± 0.3		1.51 ± 0.3		2.00 ± 0.5		0.82 ± 0.1		0.52 ± 0.1		0.44 ± 0.0		0.43 ± 0.0
20:3		0.34 ± 0.3		0.16 ± 0.2		0.29 ± 0.2		0.28 ± 0.1		0.31 ± 0.2		0.01 ± 0.1		0.02 ± 0.1		0.00 ± 0.0		0.00 ± 0.0		0.00 ± 0.0		0.00 ± 0.0		0.00 ± 0.0
20:4ω6		11.94 ± 0.9		9.29 ± 1.4		8.19 ± 0.7		9.30 ± 0.6		9.23 ± 0.6		4.98 ± 1.1		5.37 ± 1.0		9.54 ± 1.4		3.13 ± 0.2		1.37 ± 0.1		1.82 ± 0.1		4.55 ± 0.2
20:5ω3		10.18 ± 1.2		11.22 ± 1.5		9.12 ± 0.8		7.62 ± 0.6		13.97 ± 1.0		15.85 ± 1.5		15.41 ± 1.7		12.06 ± 1.4		16.72 ± 1.6		20.48 ± 0.3		18.89 ± 0.1		15.91 ± 0.6
22:3		1.24 ± 0.2		1.05 ± 0.2		1.05 ± 0.1		1.08 ± 0.1		1.34 ± 0.1		1.34 ± 0.2		1.07 ± 0.2		1.27 ± 0.1		0.89 ± 0.2		1.40 ± 0.1		0.86 ± 0.0		1.03 ± 0.1
22:4ω6		0.35 ± 0.1		0.20 ± 0.1		0.21 ± 0.1		0.23 ± 0.0		0.44 ± 0.1		0.18 ± 0.1		0.19 ± 0.1		0.31 ± 0.0		0.32 ± 0.0		0.09 ± 0.1		0.10 ± 0.1		0.23 ± 0.0
22:5ω3		6.17 ± 0.3		4.58 ± 0.9		3.82 ± 0.4		4.05 ± 0.5		4.59 ± 0.4		3.30 ± 0.6		3.25 ± 0.7		4.66 ± 0.8		2.22 ± 0.3		2.02 ± 0.2		2.02 ± 0.2		1.86 ± 0.1
22:6ω3		9.89 ± 0.7		7.98 ± 1.5		6.85 ± 0.7		6.96 ± 0.7		9.57 ± 0.2		8.28 ± 1.0		8.54 ± 1.3		10.25 ± 0.9		9.36 ± 0.6		7.93 ± 0.8		9.27 ± 0.7		8.59 ± 0.5
Sum %		54.02 ± 1.3		48.85 ± 4.3		43.16 ± 1.4		43.79 ± 1.9		50.33 ± 4.8		46.04 ± 7.6		44.97 ± 7.6		50.04 ± 6.9		42.72 ± 2.1		44.40 ± 0.5		43.37 ± 0.9		41.75 ± 0.9
DHA/EPA		0.98 ± 0.1		0.71 ± 0.1		0.75 ± 0.1		0.92 ± 0.1		0.69 ± 0.2		0.52 ± 0.7		0.55 ± 0.8		0.85 ± 0.6		0.56 ± 0.0		0.39 ± 0.0		0.49 ± 0.0		0.54 ± 0.0
Sum ω6		15.53 ± 1.0		12.75 ± 1.5		11.96 ± 0.7		13.22 ± 0.6		12.47 ± 0.9		7.64 ± 1.6		8.42 ± 1.5		12.78 ± 1.9		5.77 ± 0.1		3.56 ± 0.5		4.03 ± 0.2		7.07 ± 0.2
Sum ω3		28.79 ± 2.2		27.67 ± 3.6		23.22 ± 1.9		21.54 ± 1.6		31.35 ± 2.1		32.99 ± 4.0		32.16 ± 4.4		31.79 ± 3.6		33.96 ± 1.6		37.98 ± 1.0		37.11 ± 0.5		32.55 ± 0.9
ω3/ω6		1.87 ± 0.2		2.20 ± 0.4		1.95 ± 0.2		1.63 ± 0.1		2.52 ± 0.2		4.46 ± 1.0		3.91 ± 0.7		2.53 ± 0.38		5.89 ± 0.4		10.95 ± 1.8		9.22 ± 0.4		4.61 ± 0.2
Sum n.i. %		1.40 ± 0.3		2.63 ± 0.9		3.05 ± 1.2		1.61 ± 0.8		3.29 ± 0.6		3.52 ± 0.5		2.84 ± 0.3		2.10 ± 0.4		3.22 ± 0.5		3.43 ± 0.5		3.27 ± 0.0		2.55 ± 0.1
16:1ω7/16:0		0.26 ± 0.1		0.23 ± 0.1		0.20 ± 0.0		0.19 ± 0.0		0.19 ± 0.0		0.24 ± 0.0		0.23 ± 0.0		0.16 ± 0.1		0.20 ± 0.0		0.22 ± 0.0		0.21 ± 0.0		0.19 ± 0.0
Total mg g^-1^		3.63 ± 0.8		5.60 ± 1.4		5.73 ± 1.4		5.76 ± 1.7		12.89 ± 2.6		20.69 ± 7.7		20.35 ± 6.8		13.07 ± 3.3		60.29 ± 8.5		91.15 ± 25.7		81.97 ± 23.1		80.13 ± 24.8

Total amount (mg g^- 1^) of FA is also given.

**Table 2:** Relative contribution (%) of fatty acids in embryos (stages I to IV) of *Crepidula* *fornicata* .

**FA**	**February**	**May**								**July**								**September**							
	**Stage I**	**Stage I**		**Stage II**		**Stage III**		**Stage IV**		**Stage I**		**Stage II**		**Stage III**		**Stage IV**		**Stage I**		**Stage II**		**Stage III**		**Stage IV**	
Saturated																									
14:0	2.17 ± 0.3	2.48 ± 0.2		2.35 ± 0.2		2.12 ± 0.2		2.11 ± 0.2		3.38 ±0.1		3.03 ± 0.3		2.05 ± 0.1		2.05 ± 0.1		3.08 ± 0.2		3.90		3.31 ± 0.3		2.13 ± 0.4	
15:0	0.64 ± 0.1	0.47 ± 0.0		0.55 ± 0.0		0.51 ± 0.0		0.61 ± 0.2		0.58 ± 0.0		0.57 ± 0.1		0.50 ± 0.0		0.45 ± 0.1		0.63 ± 0.0		0.59		0.54 ± 0.0		0.40 ± 0.1	
16:0	17.39 ± 1.2	19.86 ± 0.3		19.11 ± 0.3		18.26 ± 0.8		18.68 ± 0.8		20.85 ± 0.6		20.95 ± 0.5		18.95 ± 1.0		15.49 ± 1.3		20.61 ± 0.4		20.32		20.39 ± 0.6		16.00 ± 1.0	
17:0	1.51 ± 0.2	0.91 ± 0.0		1.19 ± 0.0		1.24 ± 0.1		1.33 ± 0.1		1.04 ± 0.1		1.07 ± 0.1		1.14 ± 0.0		1.13 ± 0.1		1.30 ± 0.1		1.10		1.27 ± 0.1		0.57 ± 0.6	
18:0	5.93 ± 1.0	5.95 ± 0.7		6.29 ± 0.7		6.42 ± 0.5		6.31 ± 0.4		5.38 ± 0.3		5.55 ± 0.7		5.72 ± 0.3		6.87 ± 0.2		5.60 ± 0.7		5.38		6.00 ± 0.4		6.20 ± 0.4	
19:0	0.12 ± 0.0	0.00 ± 0.0		0.03 ± 0.0		0.00 ± 0.0		0.00 ± 0.0		0.00 ± 0.0		0.00 ± 0.0		0.00 ± 0.0		0.00 ± 0.0		0.00 ± 0.0		0.00		0.00 ± 0.0		0.00 ± 0.0	
20:0	0.38 ± 0.4	0.46 ± 0.2		0.31 ± 0.1		0.33 ± 0.1		0.24 ± 0.2		0.16 ± 0.1		0.18 ± 0.1		0.15 ± 0.2		0.06 ± 0.1		0.08 ± 0.1		0.33		0.46 ± 0.1		0.32 ± 0.3	
22:0	0.00 ± 0.0	0.40 ± 0.1		0.12 ± 0.1		0.03 ± 0.0		0.04 ± 0.1		0.07 ± 0.1		0.00 ± 0.0		0.00 ± 0.0		0.00 ± 0.0		0.09 ± 0.1		0.18		0.00 ± 0.0		0.00 ± 0.0	
Sum %	28.14 ± 0.4	30.53 ± 0.8		29.95 ± 1.0		28.90 ± 1.1		29.32 ± 0.2		31.46 ± 0.6		31.34 ± 0.7		28.50 ± 0.8		26.06 ± 1.4		31.40 ± 0.9		31.80		31.97 ± 0.7		25.62 ± 0.6	
Monounsaturated																									
15:1	0.04 ± 0.1	0.07 ± 0.1		0.05 ± 0.0		0.06 ± 0.0		0.04 ± 0.1		0.04 ± 0.1		0.08 ± 0.1		0.06 ± 0.1		0.15 ± 0.1		0.07 ± 0.1		0.06		0.04 ± 0.1		0.07 ±0.1	
16:1ω5	0.03 ± 0.1	0.00 ± 0.0		0.09 ± 0.1		0.16 ± 0.1		0.25 ± 0.2		0.09 ± 0.1		0.23 ± 0.2		0.00 ± 0.0		0.00 ± 0.0		0.10 ± 0.1		0.30		0.28 ± 0.2		0.31 ± 0.3	
16:1ω7	3.38 ± 0.1	4.29 ± 0.1		3.50 ± 0.1		3.26 ± 0.1		2.77 ± 0.1		4.35 ± 0.1		4.00 ± 0.1		3.01 ± 0.2		1.29 ± 0.9		3.84 ± 0.1		3.99		4.15 ± 0.1		2.41 ± 0.0	
16:1ω9	0.62 ± 0.1	0.51 ± 0.0		0.61 ± 0.1		0.66 ± 0.1		0.37 ± 0.3		0.66 ± 0.0		0.60 ± 0.1		0.60 ± 0.0		0.83 ± 1.2		0.60 ± 0.0		0.54		0.28 ± 0.2		0.38 ± 0.1	
17:1	0.91 ± 0.4	0.62 ± 0.5		0.78 ± 0.4		0.99 ± 0.3		1.28 ± 0.6		0.62 ± 0.4		0.61 ± 0.5		1.51 ± 0.3		4.52 ± 0.7		1.05 ± 0.3		0.26		1.01 ± 0.3		2.78 ± 0.3	
18:1ω11	0.20 ± 0.1	0.33 ± 0.1		0.31 ± 0.1		0.25 ± 0.0		0.11 ± 0.1		0.36 ± 0.1		0.33 ± 0.0		0.26 ± 0.0		0.16 ± 0.1		0.28 ± 0.0		0.21		0.25 ± 0.0		0.30 ± 0.0	
18:1ω5	0.32 ± 0.1	0.27 ± 0.0		0.33 ± 0.0		0.30 ± 0.0		0.19 ± 0.1		0.20 ± 0.0		0.25 ± 0.0		0.16 ± 0.1		0.11 ± 0.1		0.24 ± 0.0		0.13		0.24 ± 0.0		0.22 ± 0.0	
18:1ω7	3.72 ± 0.4	4.96 ± 0.3		4.05 ± 0.1		3.82 ± 0.3		3.53 ± 0.2		4.32 ± 0.2		4.36 ± 0.5		4.01 ± 0.4		3.01 ± 0.2		4.31 ± 0.1		4.15		3.45 ± 0.1		3.24 ± 0.3	
18:1ω9	3.32 ± 0.2	2.29 ± 0.2		2.82 ± 0.4		3.59 ± 0.3		3.25 ± 0.4		2.36 ± 0.2		2.51 ± 0.3		2.63 ± 0.3		2.30 ± 0.2		2.93 ± 0.0		2.48		2.85 ± 0.4		2.70 ± 0.2	
20:1ω9	3.86 ± 0.6	3.13 ± 0.3		3.87 ± 0.3		4.09 ± 0.5		4.86 ± 0.1		2.58 ± 0.1		3.34 ± 0.3		4.49 ± 0.7		6.69 ± 0.4		3.28 ± 0.2		3.25		4.74 ± 0.1		6.02 ± 0.2	
20:1	1.25 ± 0.2	0.64 ± 0.0		0.92 ± 0.1		1.27 ± 0.2		1.38 ± 0.2		0.66 ± 0.0		0.77 ± 0.1		0.94 ± 0.1		0.70 ± 0.0		0.65 ± 0.0		0.71		0.66 ± 0.1		1.13 ± 0.1	
Sum %	17.65 ± 1.0	17.10 ± 0.4		17.31 ± 0.3		18.46 ± 0.7		18.02 ± 1.3		16.22 ± 0.6		17.08 ± 0.5		17.66 ± 0.1		19.77 ± 0.5		17.35 ± 0.3		16.08		17.95 ± 0.5		19.55 ± 0.8	
Branched																									
15:0*a*	0.01 ± 0.0	0.00 ± 0.0		0.02 ± 0.0		0.00 ± 0.0		0.04 ± 0.1		0.00 ± 0.0		0.00 ± 0.0		0.00 ± 0.0		0.00 ± 0.0		0.00 ± 0.0		0.00		0.00 ± 0.0		0.00 ± 0.0	
15:0*i*	0.23 ± 0.0	0.06 ± 0.0		0.12 ± 0.0		0.11 ± 0.0		0.24 ± 0.0		0.19 ± 0.0		0.20 ± 0.0		0.05 ± 0.1		0.00 ± 0.0		0.28 ± 0.1		0.26		0.21 ± 0.0		0.16 ± 0.0	
16:0*i*	1.25 ± 0.1	0.58 ± 0.1		0.78 ± 0.1		0.86 ± 0.1		1.08 ± 0.1		0.93 ± 0.1		1.00 ± 0.2		0.80 ± 0.1		0.57 ± 0.1		1.06 ± 0.0		0.88		0.80 ± 0.0		0.39 ± 0.4	
	17:0*a*	2.39 ± 0.2	1.64 ± 0.2	1.64 ± 0.1		1.78 ± 0.2		2.14 ± 0.1		1.59 ± 0.1		1.83 ± 0.3		1.50 ± 0.1		1.19 ± 0.2		1.92 ± 0.1		1.62		1.43 ± 0.1		1.21 ± 0.3	
	17:0*i*	4.38 ± 0.5	2.26 ± 0.3	2.87 ± 0.2		3.04 ± 0.4		3.78 ± 0.2		2.96 ± 0.2		3.28 ± 0.4		2.79 ± 0.2		2.27 ± 0.1		3.69 ± 0.1		3.08		2.73 ± 0.1		2.29 ± 0.5	
	Sum %	8.27 ± 0.8	4.53 ± 0.5	5.43 ± 0.4		5.78 ± 0.7		7.29 ± 0.3		5.68 ± 0.3		6.31 ± 0.9		5.14 ± 0.4		4.04 ± 0.3		6.95 ±0.2		5.84		5.17 ± 0.3		4.05 ± 1.1	
Polyunsaturated																									
16:2		0.17 ± 0.1	0.00 ± 0.0		0.09 ± 0.1		0.19 ± 0.2		0.23 ± 0.2		0.00 ± 0.0		0.00 ± 0.0		0.00 ± 0.0		0.05 ± 0.1		0.00 ± 0.0		0.00		0.00 ± 0.0		0.00 ± 0.0
16:3		1.12 ± 0.8	0.95 ± 0.5		0.77 ± 0.1		0.97 ± 0.2		1.18 ± 0.5		0.93 ± 0.3		0.91 ± 0.3		0.68 ± 0.5		0.53 ± 0.2		0.66 ± 0.2		1.39		1.81 ± 0.2		0.96 ± 1.0
18:2ω6		2.13 ± 0.2	1.50 ± 0.1		1.84 ± 0.2		1.81 ± 0.2		2.18 ± 0.2		1.86 ± 0.1		1.88 ± 0.1		2.22 ± 0.3		2.80 ± 0.3		1.98 ± 0.0		1.79		2.04 ± 0.1		2.42 ± 0.4
18:3ω3		1.56 ± 0.2	1.49 ± 0.0		1.61 ± 0.1		1.44 ± 0.1		1.10 ± 0.1		1.71 ± 0.1		1.51 ± 0.3		1.53 ± 0.1		1.20 ± 0.2		1.49 ± 0.1		1.20		1.06 ± 0.0		1.18 ± 0.3
18:3ω6		0.19 ± 0.0	0.59 ± 0.6		0.05 ± 0.1		0.08 ± 0.1		0.04 ± 0.1		0.26 ± 0.0		0.19 ± 0.0		0.00 ± 0.0		0.00 ± 0.0		0.32 ± 0.0		0.26		0.20 ± 0.0		0.00 ± 0.0
18:4ω3		4.10 ± 0.7	6.05 ± 0.3		4.94 ± 0.1		4.03 ± 0.4		2.84 ± 0.2		5.21 ± 0.4		4.49 ± 0.8		4.53 ± 0.4		4.34 ± 0.6		4.70 ± 0.1		3.84		2.86 ± 0.1		3.44 ± 1.3
20:2		0.82 ± 0.1	0.52 ± 0.1		0.70 ± 0.1		0.83 ± 0.1		0.94 ± 0.2		0.44 ± 0.0		0.58 ± 0.1		0.96 ± 0.2		1.43 ± 0.2		0.43 ± 0.0		0.50		0.80 ± 0.0		1.16 ± 0.1
20:3		0.00 ± 0.0	0.00 ± 0.0		0.00 ± 0.0		0.00 ± 0.0		0.00 ± 0.0		0.00 ± 0.0		0.00 ± 0.0		0.00 ± 0.0		0.00 ± 0.0		0.00 ± 0.0		0.00		0.00 ± 0.0		0.00 ± 0.0
20:4ω6		3.13 ± 0.2	1.37 ± 0.1		1.88 ± 0.3		2.50 ± 0.3		3.76 ± 0.5		1.82 ± 0.1		2.04 ± 0.2		2.29 ± 0.3		4.97 ± 2.4		4.55 ± 0.2		5.08		6.26 ± 0.1		5.74 ± 2.8
20:5ω3		16.72 ± 1.6	20.48 ± 0.3		19.84 ± 0.7		19.01 ± 0.2		17.19 ± 1.2		18.89 ± 0.1		18.75 ± 0.6		18.91 ± 0.9		16.85 ± 0.5		15.91 ± 0.6		15.81		15.63 ± 0.8		19.01 ± 0.9
22:3		0.89 ± 0.2	1.40 ± 0.1		1.06 ± 0.2		0.96 ± 0.1		0.78 ± 0.1		0.86 ± 0.0		0.85 ± 0.1		0.94 ± 0.1		0.79 ± 0.3		1.03 ± 0.1		1.28		1.11 ± 0.0		0.84 ± 0.0
22:4ω6		0.32 ± 0.0	0.09 ± 0.1		0.14 ± 0.1		0.21 ± 0.0		0.26 ± 0.0		0.10 ± 0.1		0.12 ± 0.1		0.23 ± 0.0		0.08 ± 0.1		0.23 ± 0.0		0.27		0.21 ± 0.0		0.09 ± 0.1
22:5ω3		2.22 ± 0.3	2.02 ± 0.2		2.13 ± 0.2		2.58 ± 0.2		2.98 ± 0.2		2.02 ± 0.2		2.23 ± 0.2		3.11 ± 0.4		3.82 ± 0.3		1.86 ± 0.1		2.38		3.25 ± 0.3		4.18 ± 0.7
22:6ω3		9.36 ± 0.6	7.93 ± 0.8		8.44 ± 0.0		8.65 ± 0.7		9.14 ± 1.7		9.27 ± 0.7		8.79 ± 0.5		10.36 ± 0.2		9.95 ± 0.6		8.59 ± 0.5		10.44		7.32 ± 0.3		9.06 ± 1.3
Sum %		42.72 ± 2.1	44.40 ± 0.5		43.49 ± 0.1		43.26 ± 1.4		42.62 ± 3.0		43.37 ± 0.9		42.34 ± 1.7		45.76 ± 0.4		46.82 ± 1.9		41.75 ± 0.9		44.24		42.55 ± 1.3		48.07 ± 1.2
DHA/EPA		0.56 ± 0.0	0.39 ± 0.0		0.43 ± 0.0		0.46 ± 0.0		0.53 ± 0.1		0.49 ± 0.0		0.47 ± 0.0		0.55 ± 0.0		0.59 ± 0.0		0.54 ± 0.0		0.66		0.47 ± 0.0		0.47 ± 0.1
Sum ω6		5.77 ± 0.1	3.56 ± 0.5		3.91 ± 0.5		4.59 ± 0.3		6.25 ± 0.7		4.03 ± 0.2		4.23 ± 0.4		4.73 ± 0.6		7.85 ± 2.3		7.07 ± 0.2		7.40		8.71 ± 0.1		8.25 ± 3.1
Sum ω3		33.96 ± 1.6	37.98 ± 1.0		36.95 ± 0.3		35.72 ± 1.1		33.25 ± 2.8		37.11 ± 0.5		35.77 ± 1.3		38.43 ± 1.1		36.17 ± 1.9		32.55 ± 0.9		33.67		30.12 ± 1.2		36.87 ± 3.0
ω3/ω6		5.89 ± 0.4	10.95 ± 1.8		9.58 ± 1.2		7.81 ± 0.5		5.39 ± 0.8		9.22 ± 0.4		8.53 ± 0.9		8.28 ± 1.3		4.99 ± 1.3		4.61 ± 0.2		4.55		3.46 ± 0.2		5.34 ± 2.4
Sum n.i. %		3.22 ± 0.5	3.43 ± 0.5		3.82 ± 0.5		3.60 ± 0.1		2.76 ± 0.8		3.27 ± 0.0		2.92 ± 0.3		2.95 ± 0.2		3.31 ± 0.3		2.55 ± 0.1		2.04		2.36 ± 0.3		2.72 ± 0.2
16:1ω7/16:0		0.20 ± 0.0	0.22 ± 0.0		0.18 ± 0.0		0.18 ± 0.0		0.15 ± 0.0		0.21 ± 0.0		0.19 ± 0.0		0.16 ± 0.0		0.09 ± 0.1		0.19 ± 0.0		0.2		0.20 ± 0.0		0.15 ± 0.0

Values are means ± standard deviations

**Figure 3:**
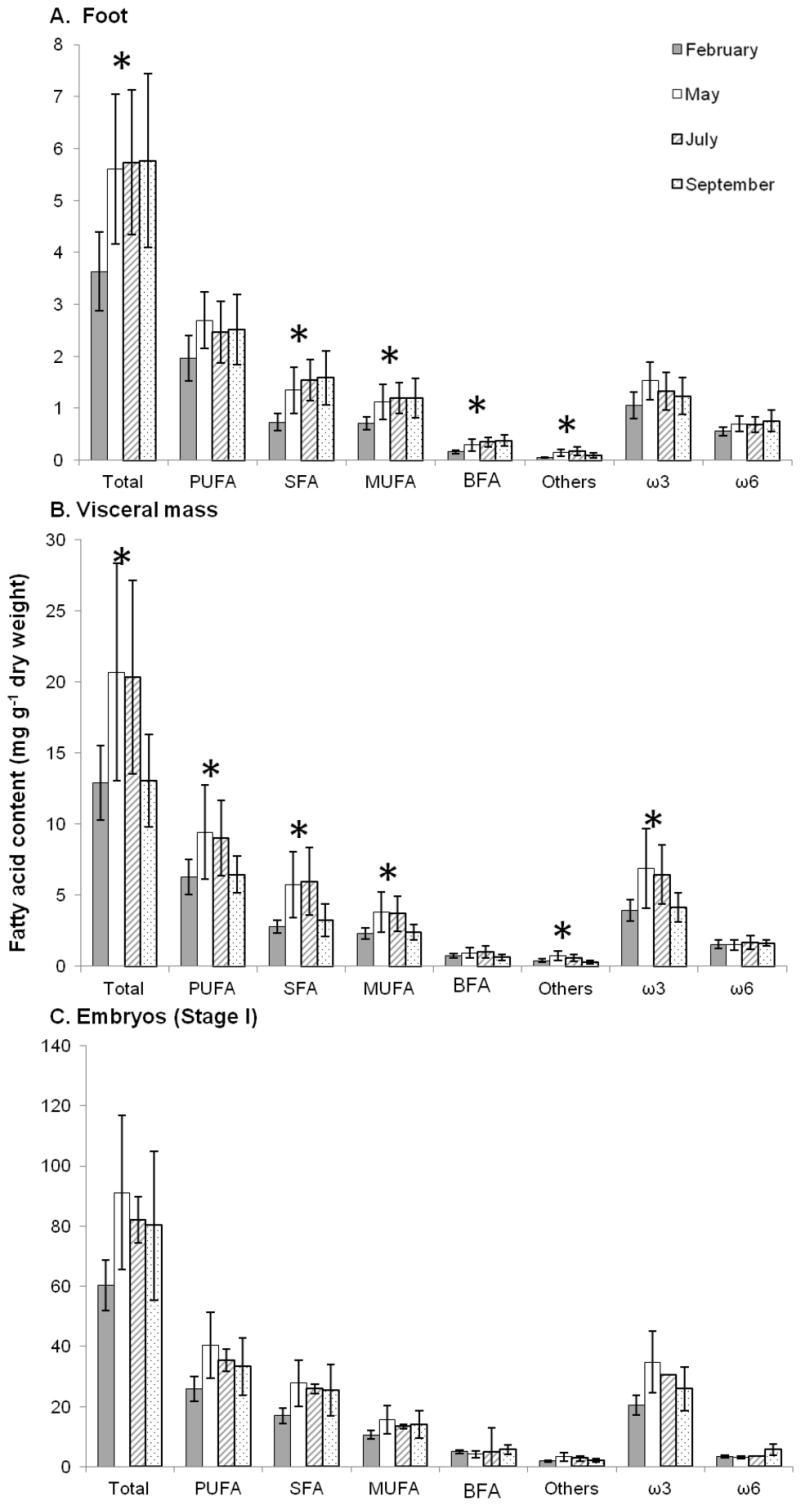
Seasonal variations of fatty acid content of females and embryos (stage I). Seasonal variations of fatty acid content (Total FA, PUFA, SFA, MUFA, BFA, Others, ω3 and ω6) at four periods of the reproductive season of *Crepidula* *fornicata* (February, May, July and September). A: foot; B: visceral mass; C: embryos (stage I). Histograms represent means and error bars correspond to standard deviations. Black stars represent significant differences between periods (Kruskal-Wallis test, *p* < 0.05).

Total fatty acid concentrations were different between the female tissues and embryos (Table 1, Figure 3), ranging from 5.41 ± 1.6 mg g^-1^ dry weight (*n*=37) in the foot to 75.47 ± 24.5 mg g^-1^ dry weight (*n*=13) in stage I embryos.

Seasonal variations in FA contents for each class of FA were tested with the non-parametric Kruskal-Wallis test (Figure 3). Except for PUFA, ω3 and ω6, all FA classes in foot samples showed a significant difference between dates (*p*<0.05). For total FA, SFA, MUFA and BFA, foot content was significantly higher in May, July and September than in February (SNK post-hoc test; *p*<0.01). In the visceral mass, except for BFA and ω6, all FA classes showed a significant difference between dates (*p*<0.05), with significantly higher total FA, PUFA (especially ω3), SFA, and MUFA concentrations in May and July than in February and September (SNK post-hoc test, p<0.05). In stage I embryos, although values were variable, no significant differences between dates were observed (*p*>0.05).

**Figure 4:**
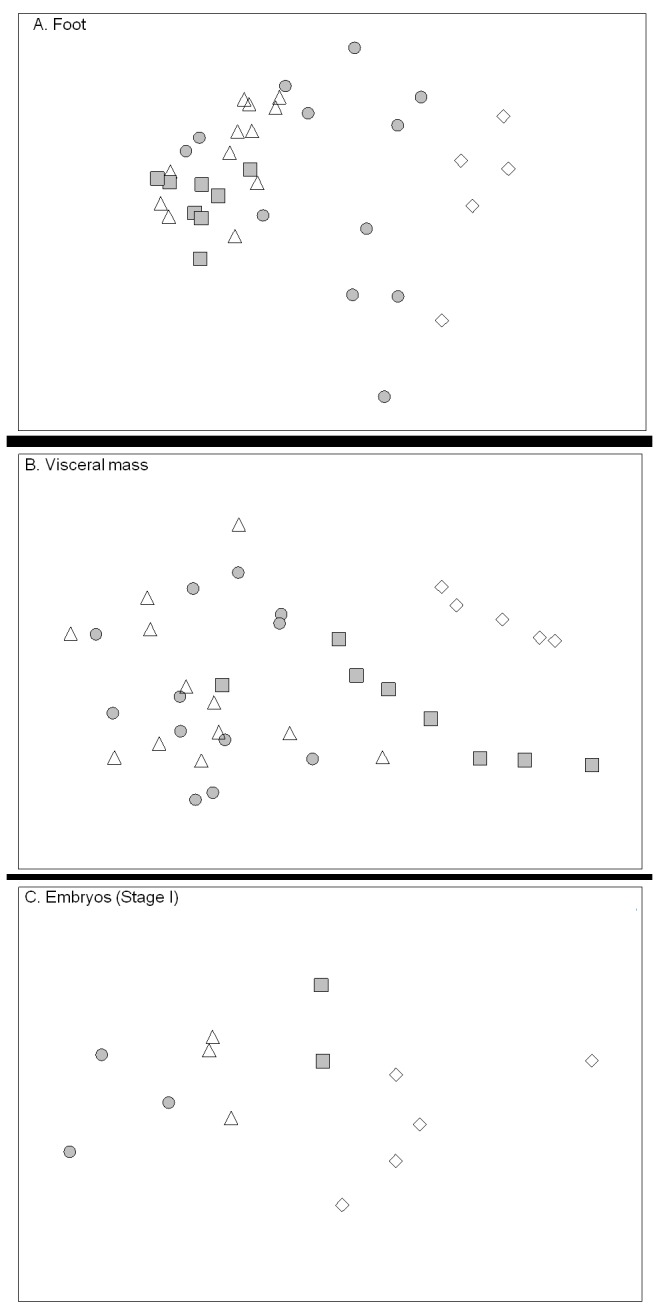
n-MDS ordination plot of females and embryos (stage I). n-MDS ordination plot of Bray-Curtis similarities based on FA composition of A: foot, B: visceral mass, and C: embryos at stage I of *Crepidula* *fornicata* . The degree of stress (0.13, 0.1, 0.04, for foot, visceral mass, and embryos at stage I, respectively) is within the range of values recommended by Clarke and Warwick [52] for robust grouping. Symbols corresponded to replicates collected in February (open diamond), May (filled circle), July (open triangle), and September (filled square).

FA composition (relative abundance, in %) of foot tissues were statistically different between sampling dates (one-way ANOSIM, *p*<0.05). Hierarchical clustering (data not shown) and n-MDS ordination (Figure 4A) clearly demonstrated a grouping of samples based on their FA composition: 1) February and May (similar at 84%), and 2) May, July and September (similar at 88%). For visceral mass, one-way ANOSIM showed significant differences in FA composition between sampling dates (*p*<0.05), which were well separated in the hierarchical clustering (data not shown) and n-MDS ordination (Figure 4B) which grouped samples from February and September (similar at 88%), and samples from May and July (similar at 88.5%). Except for May and July which showed a strong intra-group variability (R=0.121), the R statistic of other groups was between 0.54 and 1 which indicated a weak intra-group variability. Similarly for stage I embryos, n-MDS ordination (Figure 4C) and hierarchical clustering (data not shown) separated the samples into two groups at the 89% similarity level: February and September (similar at 91%) and May and July (similar at 91%).

**Figure 5:**
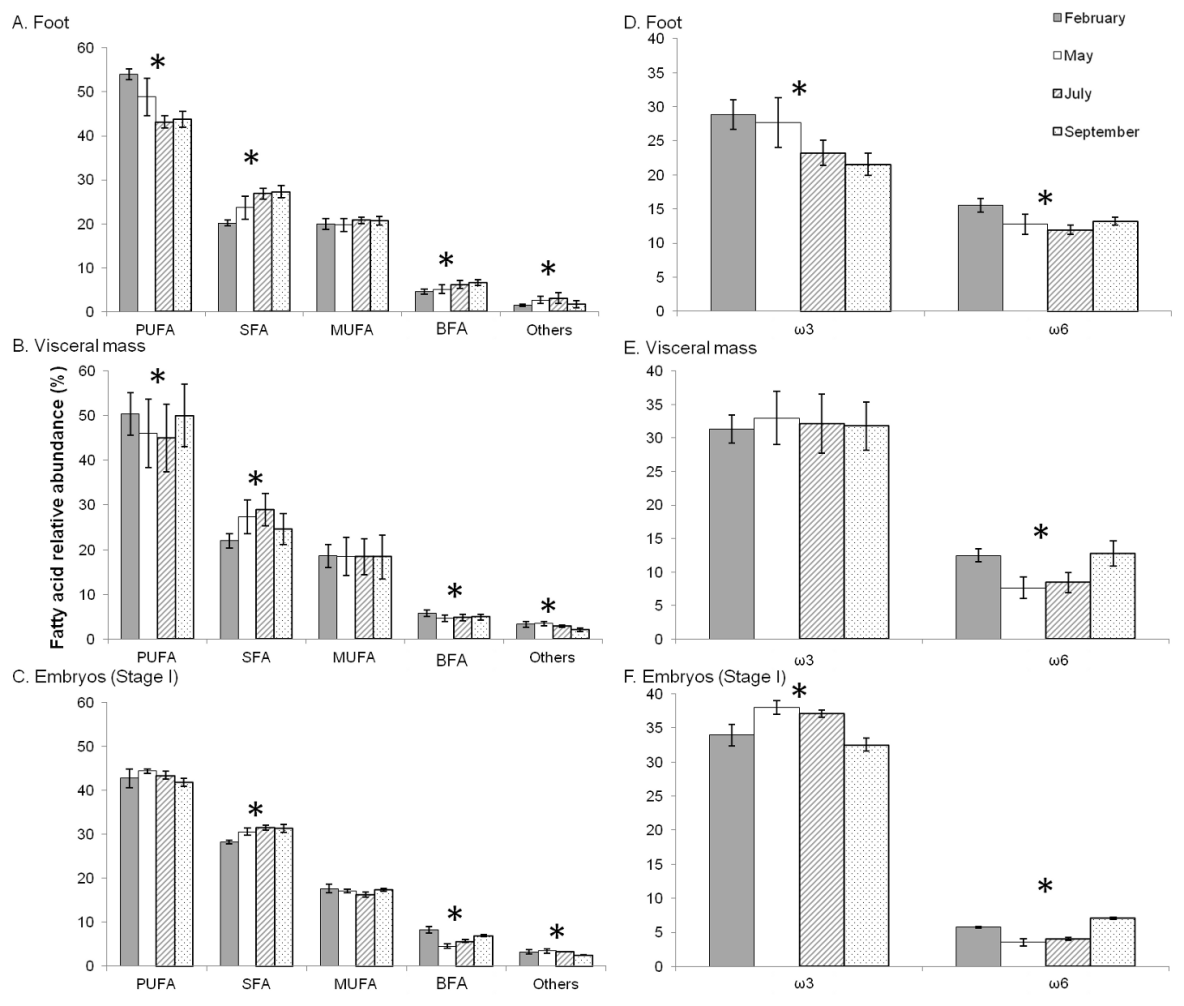
Seasonal variations of relative abundance of FA of females and embryos (stage I). Seasonal variations of relative abundance of different classes of FA (PUFA, SFA, MUFA, BFA, Others, ω3 and ω6) at four periods of the reproductive season of *Crepidula* *fornicata* (February, May, July, and September). A and D: foot; B and E: visceral mass; and C and F: embryos (stage I). Histograms represent means and error bars correspond to standard deviations. Black stars represent significant differences between periods (Kruskal-Wallis test, *p* < 0.05).

For each class of FA, differences in relative abundance between sampling dates were tested with the non-parametric Kruskal-Wallis test. In the foot, except for MUFA, all FA classes showed significant temporal differences (*p*<0.01; Figure 5A and 5D), particularly with a significant increase in SFA contribution and a significant decrease in PUFA contribution from February to September (SNK post hoc test; *p*<0.01, except between July and September where *p*>0.05). In visceral mass, except for MUFA and ω3, all FA classes showed significant temporal differences (*p*<0.05; Figure 5B and 5E), with a significantly higher SFA contribution (SNK post-hoc test; *p*<0.01, except between May and July where *p*>0.05) and a significantly lower PUFA contribution (due to a significant decrease of ω6) in May and July (SNK post-hoc test; *p*<0.01, except between May and July (*p*>0.05) and between February and September (*p*>0.05)). In stage I embryos, except for PUFA and MUFA, all FA classes showed a significant temporal difference (*p*<0.05; Figure 5C and 5F). They showed a lower contribution of SFA in February (SNK post-hoc test; *p*<0.01, no significant difference being observed between May, July and September (*p*>0.05)). These differences in the relative abundance (%) of FA between sampling dates in foot, visceral mass, and embryos at stage I were mostly due to the PUFA 18:4ω3, 20:5ω3 (EPA), 20:4ω6 (AA), and 22:6ω3 (DHA), the SFA 16: 0, the MUFA 17:1, and the BFA 17: 0*iso* (SIMPER analysis).

Altogether, the above results showed temporal variations in both FA content and composition of female tissues and embryos (stage I). In particular, they showed different compositions in May-July and February-September.

**Figure 6:**
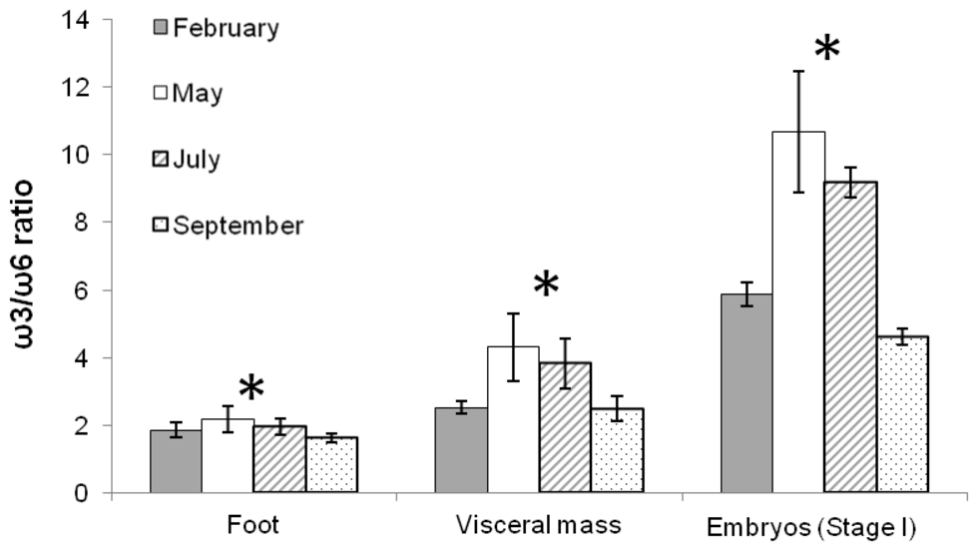
Seasonal variations of the quality index of females and embryos (stage I). Seasonal variations of the ω3/ω6 ratio at four periods of the reproductive season of *Crepidula* *fornicata* (February, May, July, and September) in foot, visceral mass, and embryos (stage I). Histograms represent means and error bars correspond to standard deviations. Black stars represent significant differences between periods (Kruskal-Wallis test, *p* < 0.05).

As a result of variations in abundance of individual FA, variations in the ω3/ω6 ratio were also observed between tissues (Kruskal-Wallis test, *p* <0.01): it was approximately twice higher in visceral mass compared to foot, and twice higher in early embryos than in visceral mass (Figure 6). Within each sample type (foot, visceral mass and stage I embryos), the ω3/ω6 ratio varied between sampling dates (Kruskal-Wallis test, *p*<0.05) (Figure 6). It was almost twice higher in May and July (middle of the reproductive season) than in February and September (beginning and end of the reproductive season). ω3 contribution in the foot decreased from 28.79% ± 2.2% in February to 21.54% ± 1.6% (*n*=37) in September (SNK post-hoc test, *p*<0.01); while ω6 contribution was higher in February (15.53% ± 1.0%, *n*=5) than at the three other dates (12.57% ± 1.2%, *n*=32) (SNK post-hoc test, *p*<0.01; Figure 5D). As a consequence, ω3/ω6 varied from 1.63 ± 0.1 to 2.20 ± 0.4 (*n*=37; Figure 6). In the visceral mass, ω3/ω6 ratios were 2.53 ± 0.3 (*n*=13) and 4.18 ± 0.9 (*n*=24) in February and September, and in May and July, respectively (Figure 6), with ω3 remaining stable (32.24 ± 2.8%; *n*=37) at the four occasions (Kruskal-Wallis test, *p* >0.05; Figure 5E); consequently, changes in the ω3/ω6 values were only due to changes in ω6 value which was 8.03 ± 1.3% (*n*=24) in the middle of the reproductive season and 12.66 ± 1.4% (*n*=13) at the beginning and at the end of the reproductive season (SNK post-hoc test; *p*<0.01). In early embryos, the ratio was 5.52 ± 0.7 (*n*=7) at the beginning and at the end of the reproductive season and 10.08 ± 1.7 (*n*=6) in the middle of the reproductive season (Figure 6). ω3 contributions varied significantly between 37.54 ± 1.0% (*n*=6) in the middle of the reproductive season and 33.55 ± 1.7% (*n*=7) at the beginning and at the end of the reproductive season (SNK post-hoc test; *p*<0.01); ω6 contributions varied between 6.14 ± 0.7% (*n*=7) for February and September and 3.80 ± 0.5% (*n*=6) for May and July (SNK post-hoc test; *p*<0.01; Figure 5F), and were half than those measured in the visceral masses.

To summary, the above results showed that female tissues and embryos at stage I have a higher ω3/ω6 ratio in May and July as compared to February and September.

### Changes in fatty acid composition during embryo development

**Figure 7:**
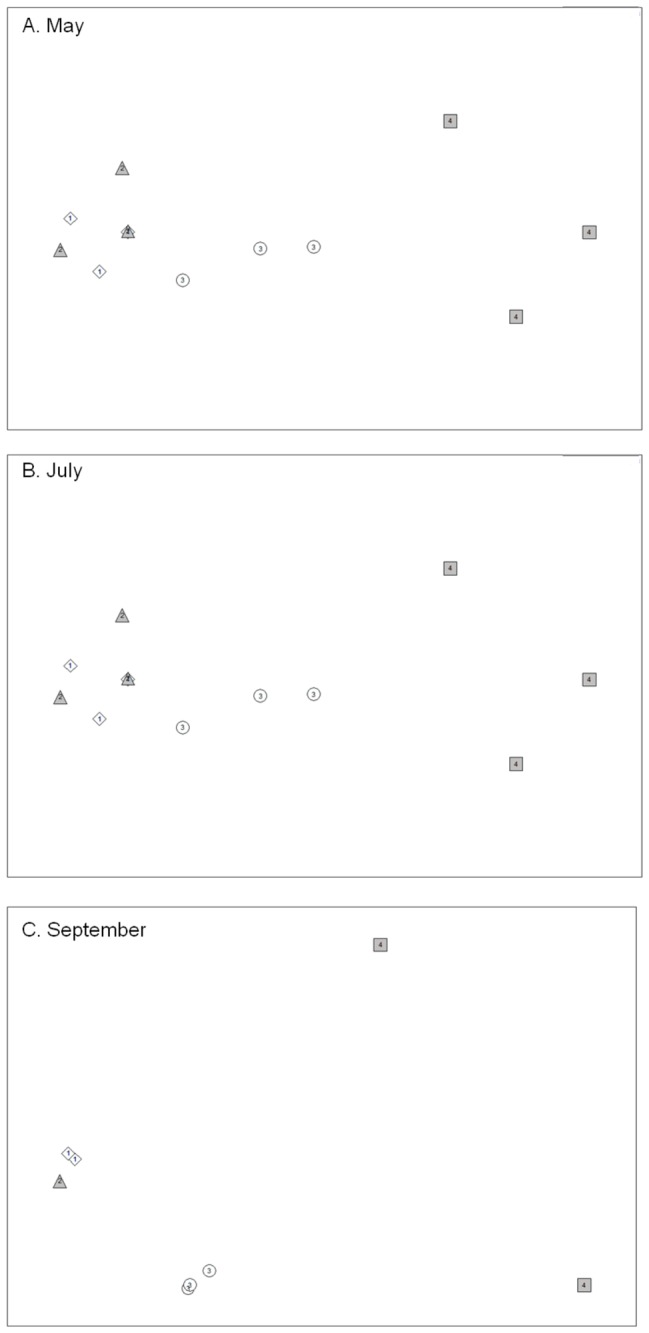
n-MDS ordination plot of embryos at stages I to IV. n-MDS ordination plot of Bray-Curtis similarities based on FA composition of embryos (stage I to IV) of *Crepidula* *fornicata* . A: May; B: July; and C: September. The degree of stress (0.05, 0.03, and 0.01, for May, July, and September, respectively) is within the range of values recommended by Clarke and Warwick [52] for robust grouping. Symbols corresponded to replicates of stages I (1; open diamond), II (2; filled triangle), III (3; open circle), and IV (4; filled square).

The n-MDS analyses of the FA composition of embryos at the four stages showed, for the three dates, differences from stage I to stage IV (Figure 7). Hierarchical clustering analysis (data not shown) confirmed this trend and demonstrated a clear separation of stages I, III, and IV. Stage II occupies an intermediate place, closer to stage I and/or III depending on the sampling date (Figure 7). Hierarchical clustering analysis further showed that stages I, II and III had similar FA composition with more than 90% similarity. Embryos at stage IV were more dissimilar (80 to 85% similarity for July and September, and more than 85% for May).

**Figure 8:**
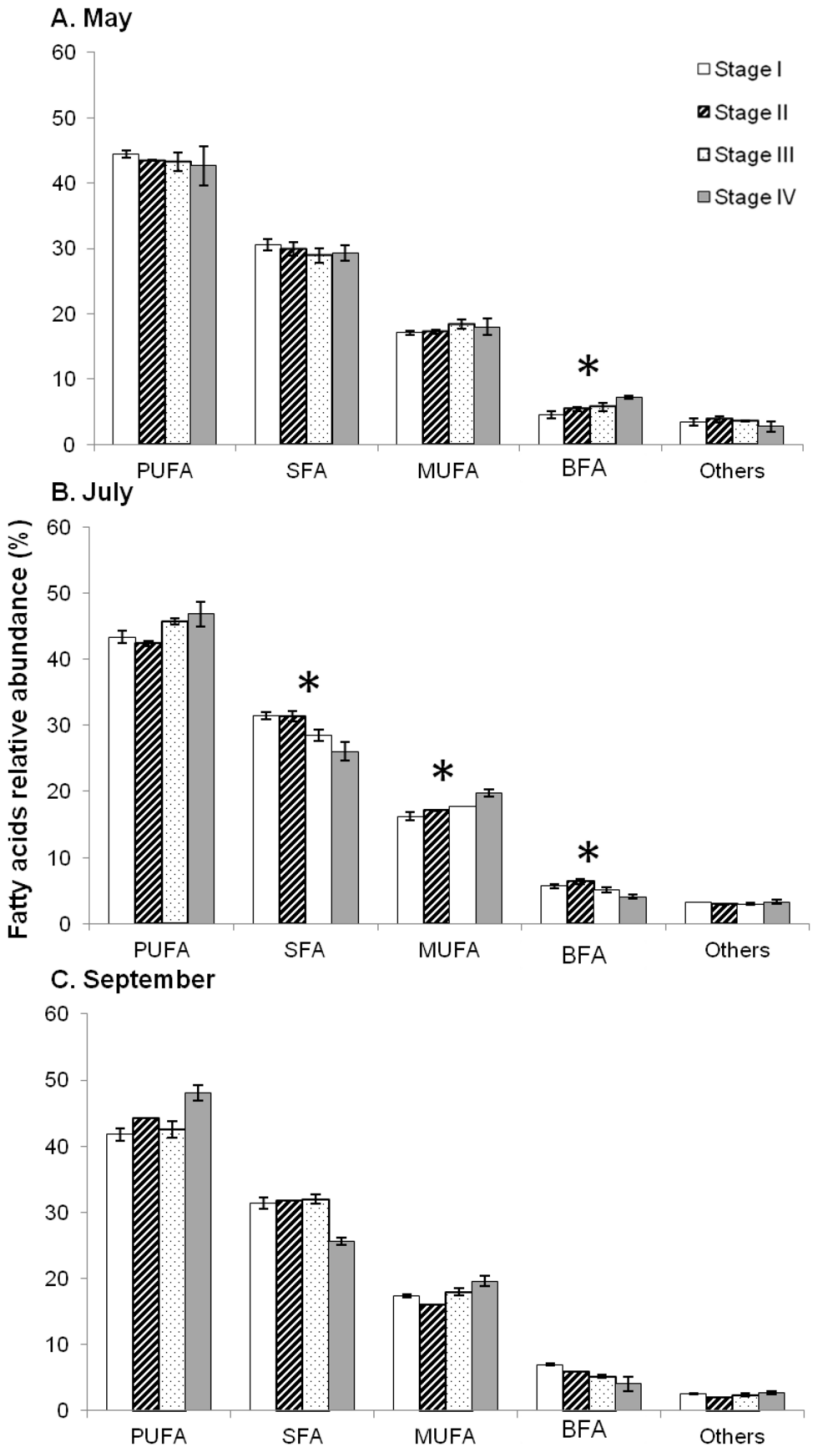
Seasonal variations of FA composition of embryos at stages I to IV. Seasonal variations of relative abundance of different classes of FA (PUFA, SFA, MUFA, BFA, and Others) at four developmental stages (I to IV) of *Crepidula* *fornicata* . A: May; B: July; and C: September. Histograms represent means and error bars correspond to standard deviations. Black stars represent significant differences between developmental stages (Kruskal-Wallis test, *p* < 0.05).

For all developmental stages, the major FA class was the PUFA (41.75 to 48.07%), followed by SFA (25.62% to 31.97%), MUFA (16.08 to 19.77%), BFA (4.04 to 7.29%) and other FA (2.04 to 3.82%) (Figure 8). The FA 14: 0, 16: 0, 18: 0, 17: 0*iso*, 16:1ω7, 18:1ω7, 20:1ω11, 18:4ω3, 20:5ω3, and 22:6ω3 were the major FA found in embryos and contributed up to 79.7-84.1% of the total FA content.

Comparisons of the relative contribution of the different FA classes (PUFA, SFA, MUFA, BFA, and others) between the four developmental stages at each sampling date with a Kruskal-Wallis test, revealed that in May, except for BFA and ω6 (*p*<0.05), there was no significant difference (*p*>0.05); in July, except for BFA, SFA and MUFA (p<0.05), all other FA classes showed no significant difference (*p*>0.05); in September, all FA classes were not significantly different (*p*>0.05) between stages. The observed differences were mostly due to the PUFA 18:4ω3, 20:5ω3 (EPA), 20:4ω6 (AA), and 22:6ω3 (DHA), the SFA 16: 0, and the MUFA 20:1ω9 (SIMPER analysis).

**Figure 9:**
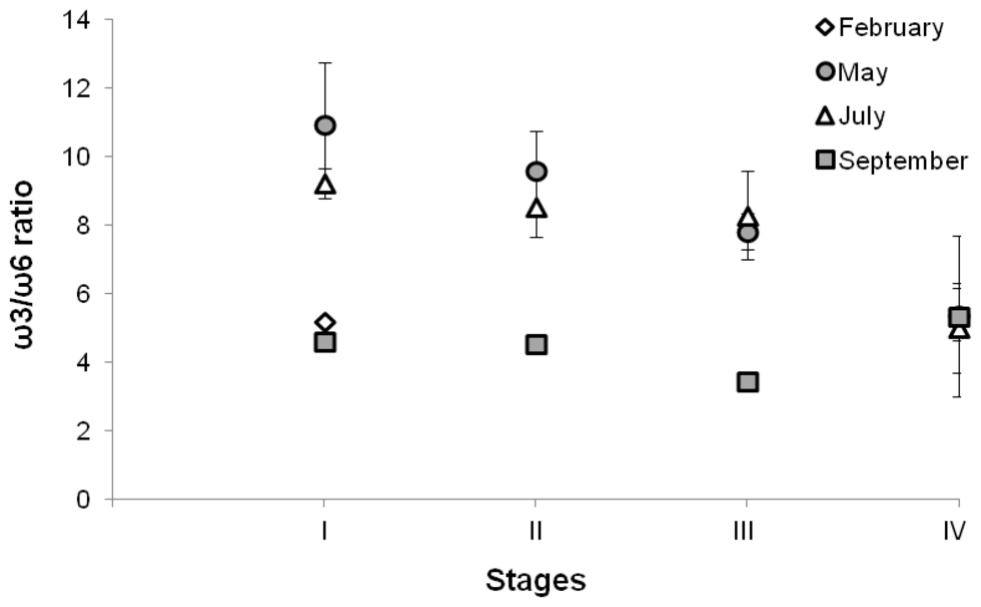
Variations of the quality index of embryos at stages I to IV. Variations of ω3/ω6 ratio in embryos at stages I to IV, at four periods of the reproductive season of *Crepidula* *fornicata* (February, May, July, and September). Points represent means and error bars correspond to standard deviations.

Looking more specifically at PUFA in May, the relative concentration of ω3 decreased from stage I to stage IV embryos (from 37.98 to 33.25%) whereas total ω6 FA increased (3.56 to 6.25%), leading to a decrease of the ω3/ω6 ratio from 11.0 to 5.4 (Figure 9). The same pattern was observed in July, with a decrease of the ω3/ω6 ratio from 9.2 to 5.0 (Figure 9). A different pattern was observed in September, with an increase of the relative concentration of ω3 (from 32.55 to 36.87%), and ω6 (from 7.07 to 8.71%) between stage I and IV, leading to an increase in the ω3/ω6 ratio between 3.5 and 5.3 (Figure 9).

### Seasonal variations in the FA composition of pelagic larvae of *C* *. fornicata*

**Figure 10:**
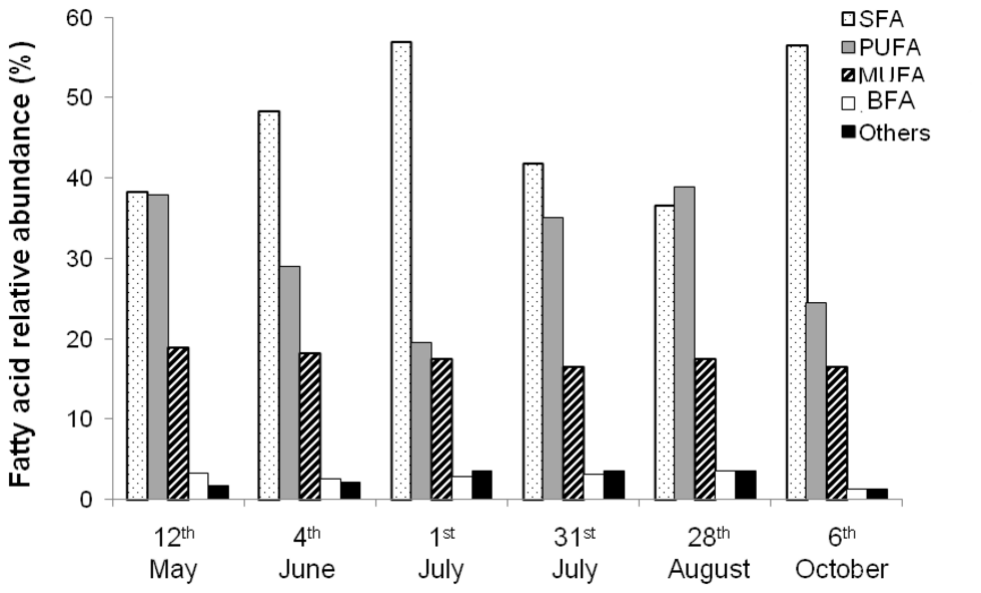
Seasonal variations of FA composition of pelagic larvae. Seasonal variations of relative abundance of different classes of FA (SFA, PUFA, MUFA, BFA, and Others) in pelagic larvae of *Crepidula* *fornicata* in 2009.

35 FA were identified in pelagic larvae (Table 3). FA composition was highly variable depending on the sampling date (Figure 10), and no clear seasonal pattern was evidenced. Total FA in larvae were mostly SFA (38.23 to 56.87%), followed by PUFA (19.48% to 37.90%), MUFA (16.40 to 18.85%), and BFA (1.29 to 3.09%) (Figure 10, Table 3), except in larvae collected on the 28^th^ August, in which PUFA dominated (38.98%). Individual FA that most contributed to the larval FA pool (between 71 to 82% of the total FA content) were the SFA 16: 0, 18: 0 and 14: 0, the PUFA 20:5ω3, 22:6ω3, 22:5ω3 and 20:4ω6, the MUFA 20:1ω9, 18:1ω9, 18:1ω7 and 16:1ω7, and the BFA 17: 0*iso*.

**Table 3:** Relative contribution (%) of fatty acids in pelagic larvae of *Crepidula* *fornicata* .

**FA**	**Larvae**					
	**12/05/2009**	**04/06/2009**	**01/07/2009**	**31/07/2009**	**28/08/2009**	**06/10/2009**
Saturated						
12:0	1.25	1.04	3.85	3.49	1.32	5.38
14:0	3.38	3.77	5.74	5.46	4.02	6.55
15:0	0.74	1.09	1.07	1.04	1.21	2.06
16:0	16.56	26.85	33.54	22.04	19.35	22.72
17:0	6.51	4.38	1.14	1.03	1.48	7.34
18:0	8.68	10.41	11.53	8.52	8.96	12.14
22:0	0.54	0.00	0.00	0.00	0.00	0.00
24:0	0.58	0.72	0.00	0.19	0.16	0.33
Sum %	38.23	48.25	56.87	41.77	36.50	56.51
Monounsaturated						
16:1ω7	2.02	4.55	2.41	3.15	2.29	1.59
16:1ω9	0.91	0.88	0.52	0.92	1.62	2.81
17:1	3.01	0.64	1.42	0.85	1.19	1.11
18:1ω7	2.53	3.99	2.42	2.95	2.62	1.24
18:1ω9	3.92	2.56	3.62	2.83	2.89	6.33
20:1ω9	5.09	4.63	6.04	4.92	5.70	2.84
20:1	1.37	0.87	0.94	0.90	1.07	0.47
Sum %	18.85	18.12	17.38	16.51	17.38	16.40
Branched						
15:0*i*	0.00	0.00	0.00	0.00	0.25	0.00
16:0*i*	0.56	0.00	0.00	0.49	0.50	0.00
17:0*a*	0.90	0.81	0.87	0.67	0.80	0.28
17:0*i*	1.82	1.69	1.91	1.93	2.05	1.01
Sum %	3.28	2.50	2.78	3.09	3.60	1.29
Polyunsaturated						
16:2	0.00	0.00	0.00	0.00	0.00	1.57
16:3	0.00	0.00	0.00	0.00	0.34	0.63
18:2ω6	1.88	1.58	1.87	1.83	1.79	2.17
18:3ω3	0.95	0.99	0.65	0.91	0.87	1.25
18:3ω6	0.52	0.00	0.00	0.00	0.00	0.84
18:4ω3	2.83	2.35	1.28	1.65	1.68	0.87
20:2	0.96	0.70	0.69	0.82	0.93	0.51
20:4ω6	3.68	1.47	1.82	3.48	5.73	3.44
20:5ω3	14.49	12.38	7.30	14.95	14.69	6.15
22:3	1.20	0.72	0.00	0.54	0.54	0.00
22:5ω3	3.40	2.71	1.85	3.09	4.09	2.35
22:6ω3	8.00	6.13	4.02	7.77	8.27	4.75
Sum %	37.90	29.02	19.48	35.03	38.92	24.52
DHA/EPA	0.55	0.50	0.55	0.52	0.56	0.77
Sum ω6	6.08	3.05	3.69	5.31	7.51	6.44
Sum ω3	29.67	24.55	15.10	28.37	29.60	15.37
ω3/ω6	4.88	8.04	4.10	5.34	3.94	2.39
Sum n.i. %	1.74	2.11	3.49	3.59	3.60	1.28
16:1ω7/16:0	0.12	0.17	0.07	0.14	0.12	0.07

**Figure 11:**
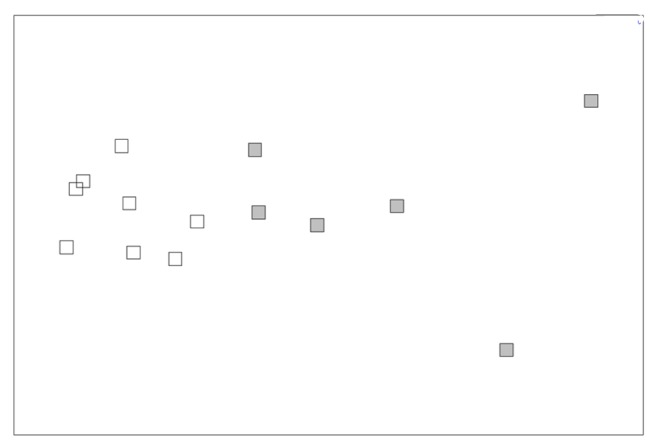
n-MDS ordination plot of embryos at stage IV and pelagic larvae. n-MDS ordination plot of Bray-Curtis similarities based on FA composition of embryos at stage IV (open squares) and pelagic larvae (filled squares) of *Crepidula* *fornicata* . The degree of stress (0.06) is within the range of values recommended by Clarke and Warwick [52] for robust grouping.

A one-way ANOSIM showed significant differences in FA composition of pelagic larvae and embryos at stage IV (*p*<0.01). The two groups were well separated after hierarchical clustering (data not shown) and n-MDS ordination (Figure 11). The n-MDS analysis (Figure 11) clearly demonstrated a grouping of the larval samples (similar at 80.2%), and of the samples of embryos at stage IV (similar at 87.3%). Differences between these two groups were mostly due to the SFA (12: 0, 14: 0, 16: 0, 17: 0 and 18: 0) and the PUFA (20:5ω3, 20:4ω6, and 22:6ω3) (SIMPER). The larval ω3/ω6 ratios (between 3.94 and 5.34) were similar to those observed in embryos at stage IV, except at two sampling dates: June (ω3/ω6 = 8.04) and October (ω3/ω6 = 2.39).

### Seasonal variations in the FA composition of POM

35 FA were identified in POM. Whatever the sampling date, depth, or pre-filtration mesh-size, POM was always dominated by SFA (56.41 to 67.56%), followed by MUFA (25.02 to 33.20%), PUFA (4.19 to 10.88%), and BFA (2.46 to 5.05%) (Table 4). The fatty acids 14: 0, 15: 0, 16: 0, 18: 0, 16:1ω7, 16:1ω9, 18:1ω7, and 18:1ω9 were the major FA found in POM samples.

**Table 4:** Relative contribution (%) of fatty acids in the particulate organic matter (POM).

**FA**	**POM**
Saturated	
12:0	2.37 ± 0.74
14:0	10.88 ± 1.83
15:0	3.80 ± 1.11
16:0	30.72 ± 2.62
17:0	2.10 ± 1.11
18:0	8.38 ± 1.58
20:0	0.16 ± 0.25
22:0	1.08 ± 0.80
24:0	1.36 ± 0.48
Sum %	60.85 ± 3.12
Monounsaturated	
15:1	1.01 ± 0.45
16:1ω5	0.36 ± 0.21
16:1ω7	7.06 ± 2.90
16:1ω9	7.35 ± 2.63
17:1	0.63 ± 0.57
18:1ω5	0.28 ± 0.21
18:1ω7	3.50 ± 0.98
18:1ω9	7.77 ± 1.43
20:1ω9	0.30 ± 0.48
Sum %	28.25 ± 2.62
Branched	
15:0*a*	1.27 ± 0.32
15:0*i*	0.93 ± 0.19
16:0*i*	0.34 ± 0.29
17:0*a*	0.22 ± 0.27
17:0*i*	1.03 ± 0.38
Sum %	3.79 ± 0.98
Polyunsaturated	
16:2	0.20 ± 0.28
16:3	0.93 ± 0.85
18:2ω6	1.22 ± 0.45
18:3ω3	0.77 ± 0.39
18:3ω6	0.26 ± 0.38
18:4ω3	0.87 ± 0.72
20:2	0.11 ± 0.19
20:4ω6	0.09 ± 0.15
20:5ω3	1.19 ± 0.85
22:3	0.37 ± 0.56
22:5ω3	0.03 ± 0.09
22:6ω3	0.42 ± 0.42
Sum %	6.46 ± 2.19
DHA/EPA	0.31 ± 0.29
Sum ω6	1.57 ± 0.75
Sum ω3	3.27 ± 2.00
ω3/ω6	2.56 ± 2.24
Sum n.i. %	0.65 ± 0.49
16:1ω7/16:0	0.23 ± 1.11

Data represent means and standard deviations of 13 samples.

## Discussion

### Seasonal variations in maternal supply to early embryos

Through its role on larval and even postlarval performance [1,3,6,54], maternal provisioning to offspring may influence dispersal and recruitment success in marine invertebrates. In species with a long reproductive period, parents may have to cope with seasonal variations of the environmental conditions, including food abundance and composition, which may in turn change resource allocation to offspring. Ultimately this may lead to seasonal changes in offspring condition (e.g. [13]), with potential consequences on recruitment success. Assessing the variations in maternal provisioning along the reproductive cycle of a given species is thus essential.

In the slipper limpet *Crepidula* *fornicata* , which reproduces from February to October, the foot and visceral mass of females showed different fatty acid compositions, as previously reported in other gastropod species [55,56]. Furthermore, both tissues showed different seasonal patterns along the reproductive period (Figure 4A and B). These differences likely highlighted the different metabolic patterns in both tissues. The foot samples clearly showed a seasonal gradient from February to July and September, with the samples from May having a high intra-group variability (Figure 4A). These latter could be divided into three subgroups: 1) samples which displayed a winter signature, similar to those of February, 2) samples which were closer to those of the summer (July and September), and 3) samples showing an intermediate position between those from February and July. In the opposite, visceral masses did not show a seasonal gradient, but a separation between females from the middle of the reproductive period (i.e. May and July) and those from the beginning and end of the reproductive period (Figure 4B).

As reported earlier by Gardner and Riley [57], unsaturated FA are dominant (up to 75% of total FA) in both female tissues. Even if we have to be cautious in comparing our results with these data - they were obtained from composite samples of whole tissues from several animals collected in November, and for three lipid classes (triglycerides, phospholipids and sterol esters) – some differences may be highlighted. First, both female tissues were characterized by high levels of C20 and C22 mono- and polyunsaturated fatty acids, most of which being essential in marine animals including molluscs, and known to play major roles in their growth, survival and reproduction ( [26,56,58], and references herein). Among them, the 20:4ω6 arachidonic acid (AA), DHA and EPA are more abundant in both foot and visceral mass than in Gardner and Riley’s samples. In particular, AA showed an interesting seasonal pattern in the visceral mass, decreasing in May and July (*ca.* 5%, compared to *ca.* 9% in February and September), most likely in relation with the reproductive activity of *C* *. fornicata* , as previously reported in abalone [56]. Indeed, AA is known to play an important role in gametogenesis as a precursor of prostaglandins (e.g. [24]). Second, as expected from their feeding regime, most marine FA biomarkers (e.g. [20]) are found in various amounts in adult tissues of *C* *. fornicata* . Indeed, adults of *C* *. fornicata* are filter-feeders which are considered omnivorous: they feed on various sizes of phytoplankton, from picoplankton to large diatoms and dinoflagellates, both directly or within particle aggregates (e.g. [59-62]), without any selection [63,64]. A noticeable difference with Gardner and Riley’s data is the absence in our study of the cetoleic acid (22:1ω11), a biomarker of predation on zooplankton (mainly copepods [65]). Feeding on zooplankton is plausible in *C* *. fornicata* especially when phytoplankton is not abundant [66], like in the winter months. These observed differences are thus likely explained by seasonal differences in food availability (less phytoplankton in November).

As food available to parents may influence the energy status of the embryos which mainly or even exclusively rely on resources provided by the mother, we studied the FA composition of the embryos. The FA composition of the early encapsulated embryos was closer to that of the visceral mass than to that of the foot. This observation is in agreement with previous results on the gastropod *Haliotis* *fulgens* by Nelson et al. [56] who showed that lipids are transferred from the hepatopancreas to the gonad to the larvae. In addition, the seasonal variations observed in the FA composition of the visceral mass were also observed in embryos at stage I, which suggested that parental feeding markedly influence the FA composition of the early embryos, as reported in other species [26,67].

Although females supplied the same proportion of total PUFA whatever the season to their embryos, we observed a preferential allocation of ω3 to early embryos in the middle of the reproductive season, as shown by the higher proportion of ω3 in embryos at stage I than in the visceral mass of the females, with the opposite trend for ω6 (Figure 5E and F). The major PUFA in early embryos and visceral masses were EPA, DHA and 18:4ω3 for ω3 and AA for ω6. Among them, EPA was accumulated in early embryos as compared to visceral mass (Table 1). Although several invertebrates, including larval stages, are able to synthesize EPA from 18:3ω3 [68-70], our observation is more likely a preferential allocation as suggested by the similarity of the relative abundance of 18:3ω3 in visceral mass and early embryo (Table 1). Specific accumulation of EPA, known to be used as an energy source for development in molluscs, has already been observed in eggs of nudibranch species, irrespective of its presence in diet [26], but also in eggs of non-molluscan taxa (*e.g.* *Daphnia* [71]).

This preferential allocation of ω3 to early embryos led to high ω3/ω6 ratios. Even if PUFA requirements for embryo development in *C* *. fornicata* are not known, it is widely recognized that the essential ω3 PUFA (especially EPA and DHA) are crucial for hatching success and larval growth and survival in bivalves [21-24,72-74], gastropods [25], and sea urchins [69,70,75]. Accumulation of ω3 over ω6 was reported for several species and the ω3/ω6 ratio was considered an index of egg quality in several fishes and invertebrates [76-79]. Following such assumptions, our results showed that in early embryos of *C* *. fornicata* , the ω3/ω6 ratio was twice higher in May and July as compared to February and September, and thus strongly suggested that early embryos had a higher quality in the middle of the reproductive season. Seasonal variations in egg quality have been demonstrated in some fish species with potential consequences on larval survival [79,80]. Although our data cannot allow us to assess such consequences, the observed changes in FA composition over the spawning season are expected to impact hatching success and larval performance in *C* *. fornicata* .

Several complementary hypotheses may explain the seasonal variations in maternal provisioning and propagule quality in *C* *. fornicata* , and more especially explain the lower quality at the beginning and end of the reproductive period. First, the food available for maternal provisioning varies seasonally, both quantitatively and qualitatively: at our study site, between September and March chlorophyll *a* concentration is low [41] with low abundances of nanoplankton and pelagic diatoms, rich in essential PUFA [81], and dominance of bacteria and cyanobacteria which are recognized as low quality food [28,81]. Second, *C* *. fornicata* females can reproduce several times during one reproductive season [31,42]. One may hypothesize that at the end of the reproductive season females will invest less energy to reproduce. This still has to be demonstrated in *C* *. fornicata* , but it has been shown that egg size and energy content may vary with brood order (e.g. [82,83]). Last, temperature might also influence maternal provisioning as has been shown through variations in egg size and hatching size in other calyptraeid species [84].

The above conclusions were drawn from a single reproductive period. To confirm these observations of the variations of maternal provisioning during the whole reproductive season would benefit from additional sampling at the same study site.

### Variations in FA composition during intracapsular development

Lipids are the major source of energy used for embryonic and larval development in many marine invertebrates, including crustaceans [85-87] and molluscs [4,88]. In *C* *. fornicata* Pandian [16] estimated that lipids provided 65.3% of the energy required for early development. We thus expected changes in the FA composition of its embryos during intracapsular development. Embryos from stage I to IV have been analyzed at three sampling dates (May, July and September) and their FA composition varied gradually during their intracapsular development (Figures 7 and 8). These changes most likely occurred through the oxidative degradation of embryonic FA as source of energy, as in *C* *. fornicata* exogenous feeding is not likely because (1) the intracapsular fluid and capsular wall only contain low amounts of lipids in many gastropod species, including calyptraeids ( [89-91], Table S1), and (2) feeding on extracapsular organic matter is not likely [35]. In addition, molluscs have a limited capacity to elongate or desaturate the PUFA 18:2ω6, 18:3ω3, 20:4ω6, 20:5ω3 and 22:6ω3 [23,26,29,92-94], suggesting that *de novo* synthesis is not likely.

During intracapsular development, we observed a decrease in all ω3 except 22:5ω3 (DPA; docosapentaenoic acid) and 22:6ω3 in May and July (where we hypothesized a preferential allocation of ω3, see above), leading to the embryos at stage IV reaching approximately the same ω3/ω6 ratio (5.23 ± 1.6; *n*=8) at the three sampling dates. This highlighted the role of energy-supply of many ω3 PUFA as reported previously [95]. In the opposite, DHA, DPA and ω6 PUFA increased in proportion, suggesting they had a major structural function. In particular, this resulted in a decrease of the EPA/AA ratio (except in September where it is low) as previously reported through embryonic development of crustaceans [96]. Similar results were observed during embryonic development of the bivalve *Patinopecten* *yessoensis* , Dall 1898, with a linear decrease in the content of the ω3 PUFA, with the exception of 22:6ω3 [97]. In several molluscan species, 22:4ω6 and 22:5ω3 (DPA) have been reported to increase during embryogenesis and to be retained by larvae during starvation [26,95]. In our study, between 1 to 4% of 22:5ω3 (DPA) were found in embryos and larvae of *C* *. fornicata* , whereas 22:4ω6 was found in small amounts (0.09% to 0.32%), only in embryos. The need for these particular FAs is, however, species-dependent. For example, *Tapes semidecussata* (= *Venerupisphilippinarum* , Reeve 1864) and *Mercenaria* *mercenaria* , Linnaeus 1758, needed 22:6ω3 (DHA) while *Crassostrea* sp., Sacco 1897, showed a fundamental need for 20:5ω3 (EPA) [95,98]. EPA has also been reported to decrease during embryonic and larval development in many bivalve species [4,26,95,99-101]. Finally, we observed a linear decrease (from approximately 5.0% to 3.0% in May, July, and September) of the MUFA 18:1ω7 which suggested a nutritive role, contrary to the observation of its linear increase during embryonic development of the pectinid *Crassadoma* *gigantea* by Whyte et al. [4], which suggested an essential structural role for this FA.

### Larval feeding and seasonal variations in fatty acid composition of the larvae

The FA composition of pelagic larvae of *Crepidula* *fornicata* was highly variable depending on the sampling date and no clear seasonal pattern emerged (Table 3). Larvae sampled at our site mostly had a small size close to the hatching size. Thus we expected these larvae to exhibit a FA composition similar to that of late embryos (stage IV). However, larvae showed markedly different compositions, mostly due to the relative contributions of SFA and PUFA. The dominance of SFA, especially the palmitic (16: 0) and stearic (18: 0) acids in pelagic larvae has also been observed in larvae of other molluscan species [56,73] and has been assumed to indicate a non-optimal condition [102], despite the dietary interest of short SFA [103,104]. Whether the pelagic larvae of *C* *. fornicata* were in sub-optimal condition is not known. Newly-hatched veligers are able to feed on particles immediately upon release. They are also able to use dissolved organic matter, but free fatty acids have not been tested [33,35]. Thus it is unlikely that larvae of *C* *. fornicata* were starving. Alternatively, the higher 16: 0 and other SFA levels may indicate that larvae were feeding on SFA-rich particulate organic matter (POM). FA composition of the POM at the sampling site was indeed dominated by SFA (with more than 50% of total FA). An explanation for high levels of SFA in POM would be that bacteria and cyanobacteria ( *Synechococcus* sp.), both rich in SFA [20,105] are a dominant component of the microbial community in these waters [106]. Cyanobacteria are well recognized as low quality food for zooplankton [28,105,107] being depleted in PUFAs and sterols [74,105,108].

In conclusion, the FA compositions of female tissues and embryos of *C* *. fornicata* varied between seasons, with embryos of higher quality in May and July (middle of the reproductive period). Our study suggested a preferential allocation of ω3 in May and July to embryos. In addition, the FA composition of embryos varied during intracapsular development (from stage I to stage IV), with different patterns depending on sampling dates. One consequence was that the latest stage reached the same quality regarding the ω3/ω6 ratio. This suggested that the excess of ω3 was consumed when reaching stage IV and that it favored only the intracapsular development. Replicating this sampling during longer term studies would allow confirming these observations. Future work might also include feeding experiments with various controlled food sources (e.g. different levels of PUFA) which would allow better understanding FA allocation to the developing embryos. Pelagic larvae sampled at our site showed FA profiles clearly different from those of embryos at stage IV, with a dominance of SFA as compared to early embryos and females. The SFA dominance might be due to availability of low quality food for larvae but this needs to be confirmed by experimental studies. Besides, the FA composition of larvae varied between the six sampling dates and further investigations are needed to link this composition to their available sources.

## Supporting Information

Table S1 **FA content (mg g^-1^) of the capsule wall of *Crepidula* *fornicata* .** Only FA representing 1% or more of the total FA content are indicated. Results were obtained from pooled capsule walls of a single female.
(DOCX)
