# Supplementary material for: Seasonal Variations in Maternal Provisioning of Crepidula fornicata (Gastropoda): Fatty Acid Composition of Females, Embryos and Larvae
Source: PLoS One. 2013 Sep 24;8(9):e75316. doi: 10.1371/journal.pone.0075316 (PMC3782457; doi:10.1371/journal.pone.0075316)
Supplement: Table S1 — FA content (mg g-1) of the capsule wall of Crepidula fornicata . Only FA representing 1% or more of the total FA content are indicated. Results were obtained from pooled capsule walls of a single female. (DOCX) [file pone.0075316.s001.docx]

**Supporting information**

**Table S1.** FA content (mg g^-1^) of the capsule wall of *Crepidula fornicata*. Only FA representing 1% or more of the total FA content are indicated. Results were obtained from pooled capsule walls of a single female.

| **FA name** | **Mass (mg g^-1^)** |
| --- | --- |
| 12:0 | 0.010 |
| 14:0 | 0.026 |
| 16:0 | 0.126 |
| 16:1**ω**9 | 0.007 |
| 16:1**ω**7 | 0.050 |
| 17:0iso | 0.006 |
| 17:0 | 0.074 |
| 18:0 | 0.038 |
| 18:1**ω**9 | 0.029 |
| 18:1**ω**7 | 0.087 |
| 18:1**ω**5 | 0.009 |
| 18:2**ω**6 | 0.009 |
| 18:4**ω**3 | 0.009 |
| 20:1**ω**9 | 0.013 |
| 20:4**ω**6 | 0.006 |
| 20:5**ω**3 | 0.030 |
| 22:6**ω**3 | 0.014 |
